# Spoken Word Segmentation in First and Second Language: When ERP and Behavioral Measures Diverge

**DOI:** 10.3389/fpsyg.2021.705668

**Published:** 2021-09-17

**Authors:** Annie C. Gilbert, Jasmine G. Lee, Kristina Coulter, Max A. Wolpert, Shanna Kousaie, Vincent L. Gracco, Denise Klein, Debra Titone, Natalie A. Phillips, Shari R. Baum

**Affiliations:** ^1^School of Communication Sciences and Disorders, McGill University, Montréal, QC, Canada; ^2^Center for Research on Brain, Language and Music, Montréal, QC, Canada; ^3^Integrated Program in Neuroscience, McGill University, Montréal, QC, Canada; ^4^Department of Psychology, Concordia University, Montréal, QC, Canada; ^5^Center for Research in Human Development, Montréal, QC, Canada; ^6^Montreal Neurological Institute, McGill University, Montréal, QC, Canada; ^7^School of Psychology, University of Ottawa, Ottawa, ON, Canada; ^8^Haskins Laboratories, Yale University, New Haven, CT, United States; ^9^Department of Psychology, McGill University, Montréal, QC, Canada

**Keywords:** speech segmentation, word segmentation, bilingualism, event-related potentials, language dominance, audiovisual integration

## Abstract

Previous studies of word segmentation in a second language have yielded equivocal results. This is not surprising given the differences in the bilingual experience and proficiency of the participants and the varied experimental designs that have been used. The present study tried to account for a number of relevant variables to determine if bilingual listeners are able to use native-like word segmentation strategies. Here, 61 French-English bilingual adults who varied in L1 (French or English) and language dominance took part in an audiovisual integration task while event-related brain potentials (ERPs) were recorded. Participants listened to sentences built around ambiguous syllable strings (which could be disambiguated based on different word segmentation patterns), during which an illustration was presented on screen. Participants were asked to determine if the illustration was related to the heard utterance or not. Each participant listened to both English and French utterances, providing segmentation patterns that included both their native language (used as reference) and their L2. Interestingly, different patterns of results were observed in the event-related potentials (online) and behavioral (offline) results, suggesting that L2 participants showed signs of being able to adapt their segmentation strategies to the specifics of the L2 (online ERP results), but that the extent of the adaptation varied as a function of listeners' language experience (offline behavioral results).

## Introduction

Listening to one's native language, it is easy to forget that speech is a continuous stream of sounds without clear breaks between words. However, the absence of such breaks becomes abundantly clear when listening to a foreign language. In such instances, we may hear little other than strings of sounds that flow into one another. Therefore, when attempting to acquire a new language, learners must not only acquire the words and sounds of a language, but they must also learn how to segment or isolate meaningful items from one another within the continuous speech stream. Studies investigating how second language (L2) listeners manage to segment L2 lexical items from the continuous signal come to different conclusions depending on factors which include the languages spoken by the participants, the experimental methodologies used, or even the populations involved. In the present paper, we investigate word segmentation across languages within French-English bilinguals from a highly bilingual environment (Montreal, Canada) using a paradigm that provides both overt (button press) and covert (electroencephalography; EEG) responses.

The literature on word segmentation has generally demonstrated that listeners can use a vast array of cues to locate word boundaries, from suprasegmental features (Lehiste, 1972; Quené, 1993; Sanders and Neville, 2003b) to phonotactics (Mehler et al., 1981; Suomi et al., 1997; McQueen, 1998; Skoruppa et al., 2015), to top-down lexical knowledge (Davis and Johnsrude, 2007). Nonetheless, native listeners of different languages seem to favor or rely mainly on specific strategies to locate word boundaries. For example, speakers of stress-timed languages (like English or Dutch; see Pike, 1945) are particularly sensitive to lexical stress, as cued by the prosodic cues of fundamental frequency, duration, and amplitude (Fry, 1955; Lieberman, 1960; Lehiste, 1976; Grimson, 1980; Beckman Mary, 1986), and rely on its regularity to locate word *onsets* in the speech stream (Cutler and Norris, 1988; Cutler and Butterfield, 1992; Cutler et al., 1997; Jusczyk et al., 1999; Mattys et al., 1999). For instance, Cutler and Norris (1988) presented native English listeners with bisyllabic pseudowords created by adding rimes (VC) to real monosyllabic words (CVCC, e.g., *mint* + “*ayve*” = *mintayve*) where the second syllable could either be produced as stressed or not (originally labeled as “strong” or “weak”). By asking participants to respond every time they heard a pseudoword beginning with a real English word, they observed that listeners had a much harder time locating the real CVCC words when the second syllable was stressed than when it was not. This result suggested that the stressed syllable had been interpreted as the onset of a new word, which essentially “removed” the final consonant of the real English word (*min*/*tayve*), significantly slowing its recognition.

On the other hand, speakers of syllable-timed languages (like French) have been found to be particularly sensitive to syllable duration and structure (Mehler et al., 1981; Cutler et al., 1986), relying on phrase-final boundaries marked by syllabic lengthening (often produced without F0 rise) to locate word *offsets* in the speech stream (Rietveld, 1980; Cutler et al., 1997; Christophe et al., 2001, 2003, 2004, 2008). Thus, when Christophe et al. (2004) presented native French listeners with sentences containing temporary segmentation ambiguities, they observed that it was easier for participants to locate specific monosyllabic words when they were presented at phrase boundaries. For example, it was easier for participants to locate the word *chat* (Engl.: *cat*) in sequences like [*le gros chat*] [*grimpait*...] (Engl.: [*the large cat*] [*was climbing*]) than in sequences like [*un grand chat grincheux*] (Engl.: [*a large grumpy cat*]), because the activation of the potentially concurrent word *chagrin* (phonetic equivalent of *chat* + *grin/grim*, Engl.: *sorrow*) was blocked by the phrase boundary, which provided a clear word offset cue.

Interestingly, while native listeners of these languages have been found to favor one segmentation strategy over another, they nonetheless remain able to use other word segmentation cues or strategies when necessary. For example, in an artificial language learning task, Tyler and Cutler (2009) showed that native English listeners use syllabic lengthening as a word offset marker in the absence of lexical stress cues. Similarly, native French listeners have been found to use phonotactic probabilities to locate word boundaries in artificial language learning tasks when the artificial language does not include prosodic cues (Tremblay et al., 2017; see also Spinelli et al., 2010, and Do Carmo-Blanco et al., 2019, for studies on the use of word-initial F0 rises by native French listeners). Thus, even though some strategies are optimal for the processing of certain languages (and therefore favored by native speakers), listeners are not bound to them and remain able to use other segmentation strategies when needed.

Of relevance here, one might wonder what happens when listeners learn an L2 that relies upon a different preferred strategy. Previous studies on L2 word segmentation have yielded equivocal results, which is not surprising given the many ways in which bilinguals can vary. For instance, earlier studies investigating L2 word segmentation among English-French bilinguals suggested that listeners learned their L1's optimal strategy (language-specific or “restricted” strategy) during infancy and continued to rely on that strategy when processing an L2, regardless of its effectiveness in the second language (Cutler et al., 1986, 2006; Otake et al., 1993; Cutler and Otake, 1994; Weber and Cutler, 2006).

However, given that listeners remain able to use other segmentation strategies when needed, one would expect them to be able to recruit these strategies in order to process their L2 efficiently, perhaps once they have attained a certain level of proficiency in the L2. Later work involving highly proficient simultaneous French-English bilinguals has supported this notion to some extent. For example, whereas Cutler et al. (1992) replicated the results described above, showing that highly proficient bilinguals do not consistently use the appropriate segmentation cue in their non-dominant language (e.g., French dominant speakers did not use lexical stress when processing English words and English dominant speakers did not use syllable structure when processing French), their results also suggested that the same highly proficient simultaneous bilinguals were able to refrain from applying their dominant language's segmentation strategy to the non-dominant language (e.g., French dominant speakers did not use syllable structure when processing English, and English dominant speakers did not use lexical stress when processing French). Thus, in cases where a dominant language strategy would prove unsuccessful for segmentation in the non-dominant language, highly proficient bilingual listeners would resort to “non-restricted” strategies or strategies that rely on “universal rather than language-specific phonological characteristics” (Cutler et al., 1992, p. 408). Such results suggest that listeners are not as constrained as previously thought, but rather seem able to adapt their segmentation strategies to a certain degree, learning to rely on non-restricted instead of restricted strategies, even if they are unable to master the new L2's restricted strategies.

Conversely, subsequent work on English-French bilinguals demonstrated that proficient L2 learners may, in fact, adopt preferred L2 segmentation strategies. Using a word spotting paradigm in which participants were asked to identify monosyllabic words embedded in longer words (e.g., locating the word *chat* [∫ a] “cat” in *chalet* [∫ alε] “cabin”), Tremblay et al. (2012) demonstrated that more proficient English L1/French L2 listeners processed lengthened syllables as word offsets in French sentences (i.e., in a French-like manner) while less proficient listeners continued to process lengthened syllables as indicative of word onsets (treating the lengthening as an acoustic cue to lexical stress, which most frequently occurs word-initially in English). These findings suggest that highly proficient English learners of L2 French have learned to use the restricted segmentation strategy of their L2 (lengthened syllable with F0 rise = phrase final = word offset) and were not simply relying on backup or nonrestricted segmentation strategies like listeners with lower L2 proficiency have been shown to do.

Of note, in this paradigm, increased French L2 proficiency was not accompanied by an adaptation in the interpretation of F0 rises that often co-occur with phrase final lengthening in French, suggesting that not all acoustic cues are equally easy to adapt. In a later study by Tremblay et al. (2017), adaptation of the interpretation of F0 patterns was observed using an artificial language learning paradigm. In this study, the investigators showed that English L1/French L2 listeners benefitted from the addition of an F0 rise on artificial word offsets, whereas the performance of monolingual English listeners remained unchanged in the context of the F0 rise. Contrary to monolingual English listeners, English L1/French L2 bilinguals interpreted the F0 rise as a word offset cue, which helped them segment artificial words from the continuous speech stream. This result suggests that experience with French, where F0 rises are associated with phrase boundaries and word offsets, influenced English-L1 listeners' interpretation of that cue, even in the context of an artificial language (i.e., not when participants were listening to French *per se*). Such findings further support the idea that experience with an L2 with a different favored segmentation strategy has an impact on listeners' use of specific acoustic cues, indicating that L2 listeners may, indeed, be able to learn to use L2-restricted segmentation strategies.

Surprisingly, increased L2 proficiency also affected the L1 restricted segmentation strategy. That is, Tremblay et al. (2017) observed that French L1/English L2 bilingual listeners benefited to a lesser degree from word final F0 rises than monolingual French listeners, suggesting that native French listeners who have had experience with English were less likely to process F0 rises as word offset cues than native French listeners without exposure to English. Such results led the authors to suggest that segmentation strategies are adaptable and are modulated to the specifics of the listeners' linguistic environment, but that bilingual listeners have only one segmentation strategy that they use in their two languages (see Tremblay et al., 2017, for a discussion of the adaptive and non-selective nature of word segmentation).

It is important to note that the studies described above relied either on word- or syllable- “spotting” paradigms, or on artificial language learning paradigms with impoverished prosody compared to real speech. Although these tasks provide insights into what listeners attend to in the speech signal, they might not reflect how listeners typically extract words from connected speech. Furthermore, all of the studies relied on overt responses by the participants, which can be influenced by factors like self-confidence and response biases (Macmillan and Creelman, 2005).

Interestingly, work using covert measures of word segmentation in sentence contexts have yielded more nuanced results, demonstrating that the phenomenon is even more complex than previously thought. For example, Tremblay et al. (2016, 2018) have shown that the prosodic system of the L1 has a significant impact on how participants adapt their segmentation strategy to their L2, regardless of L2 proficiency. Using a visual world eye-tracking paradigm, the authors showed that English, Dutch, and Korean native speakers matched on their L2 French proficiency and experience did not rely on F0 changes to the same degree to locate word offsets in French sentences. They attributed this differential use of F0 in French to the different weight attributed to F0 in the participants' L1 (see Tremblay et al., 2016; and Tremblay et al., 2018, for a discussion of perceptual assimilations in the context of word segmentation and the Prosodic-Learning Interference Hypothesis.) Thus, studies using natural sentences and covert responses suggest that L2 learners are able to adapt their segmentation strategy to the specifics of an L2, but that their ability to do so will vary as a function of their L1 and their overall L2 experience.

Taken together, the literature on bilingual or L2 word segmentation might appear to be rife with inconsistencies, but this is not surprising, given the number of ways in which the studies vary (e.g., language pairs involved, individual differences between participants, tasks used). These inconsistencies lead to a number of unresolved questions, which we addressed here. Do English-French bilingual listeners show signs of adapting their segmentation strategies to the specifics of the L2? If so, do individual differences with regards to listeners' L1 and their L2 experience (as indexed by their relative proficiency in English vs. French) have an impact on that adaptation? And finally, does the type of data collected (overt vs. covert) have an impact on the conclusions one can draw regarding segmentation strategy adaptation since they reflect different stages of processing?

To answer these questions, we designed a cross-modal (audiovisual) integration paradigm allowing for the collection of both overt behavioral responses and covert EEG data using natural English and French sentences. The task involved listening to sentences during which an illustration appeared on screen, and participants were asked to determine whether or not the illustration was related to the heard utterance. The sentences were built around syllable strings that could be segmented as either one bisyllabic word or two monosyllabic words, while the illustrations could either represent the bisyllabic segmentation, the two monosyllabic words segmentation, or something completely unrelated. Pictures appeared onscreen at the onset of the second syllable of interest and ERP averages were time-locked to the presentation of the picture.

Thus, when designing the present study, we sought to control and account for a number of relevant variables to obtain a clearer picture of L2 vs. L1 word segmentation compared to past work. For example, we elected to use a somewhat more ecological task, where participants listen to natural sentences including word segmentation ambiguities to obtain an index of *online* sentence processing. Our bilingual sample includes native speakers of both languages (English-L1 and French-L1) and all participants were tested in both languages. This has allowed us to untangle L2 learning effects (that would affect all L2 trials, regardless of the L1) from potential language-specific directional effects (learning English vs. French first). We also used a paradigm that allows for the collection of both overt (button press) and covert (EEG) responses to the same trials, in an effort to observe the range of processes that lead to the lexical segmentation decision.

The behavioral studies on L2 segmentation lead us to expect significant individual differences in language experience on overt segmentation decisions. However, on the other hand, the literature on Event-Related Potentials (ERPs) associated with speech segmentation provides fewer studies from which to draw predictions. For example, Sanders and Neville (2003a) compared ERPs to identical syllables presented within different types of English sentences and found that word-initial syllables elicited larger N100 and N200-300 components than word-medial syllables over midline and medial electrodes. Interestingly, they also found that stressed syllables elicited larger amplitude N100s and N200-300s than unstressed syllables over anterior sites. Given the different distribution of the two effects, the authors concluded that “word-onset effects were indexing speech segmentation rather than physical differences in the stimuli” (Sanders and Neville, 2003a). Of note, different results were observed when non-native listeners were tested using the same paradigm. Namely, the position of syllables within words (initial vs. medial) had no impact on the amplitude of the N100 or N200-300 of Japanese late-learners of English. Stress, on the other hand, had no impact on the amplitude of the N100, but had a significant impact on the amplitude of the N200-300, with stressed syllables yielding larger N200-300 than unstressed syllables (Sanders and Neville, 2003b). According to the authors, these results suggest that native Japanese late-learners of English do process lexical stress (as indexed by the stress-related N200-300), albeit not in a native-like manner (as indexed by the absence of N100s or N200-300 responses related to word onset), thus indicating that language experience plays a role in how listeners use L2-specific prosodic cues.

Based on these results, different predictions may be made with regard to the present design. First, regarding experimental conditions, one might expect amplitude modulations within the N100 and late N200 time-windows as a function of sentence conditions (segmented as either one bisyllabic word or two monosyllabic words) given that ERPs were time-locked to the onset of the second syllable of interest. Thus, in the one bisyllabic word condition, ERPs were time-locked to a word-medial unstressed syllable, while in the two monosyllabic words condition, ERPs were time-locked to a stand-alone monosyllabic word. Second, regarding bilingual listeners' processing of these sentences, one might expect participants' individual differences in language experience (L1 or relative language dominance) to be associated with amplitude modulations in the N100 and late N200 time-windows compared to native listeners. In the present design, if L2 listeners cannot learn to use L2-appropriate segmentation cues, one would expect French-L1 listeners to show reduced amplitude in the N100 time-window during English trials, like the English-L2 listeners of Sanders and Neville (2003b). Also, provided that French native listeners are believed to be stress-deaf by some authors, one might also expect to observe reduced amplitudes in the late N200 time-windows indicating that French-L1 listeners did not perceive lexical stress and therefore could not use it as a word onset cue (see Dupoux et al., 1997, for a discussion of French native listeners' alledge stress “deafness”).

On the other hand, if L2 listeners can indeed learn to rely on L2-specific segmentation cues, one would expect no significant differences between the ERPs of French-L1 and English-L1 listeners. Although we could not find an equivalent study investigating French, we would nonetheless expect to find significant L1 effects within the ERPs only if participants are not able to learn to use L2-specific segmentation cues.

Furthermore, the present paradigm involves the presentation of illustrations while participants are listening to sentences and requires participants to determine if the illustration is related to the sentence. In such a design, the auditory and visual streams of information must be integrated for participants to perform the task, a process that might have an impact on the observed ERPs. For example, Yin et al. (2008) asked participants to determine if visually presented Chinese ideograms were related to recordings of isolated Mandarin words while monitoring participants' brain activity. The authors observed ERP peaks in the N100 and N400 time-windows for each trial, but found significant effects of relatedness between visual and auditory stimuli only within the N400 time-window (N100 peak amplitude remained stable across conditions, which can be interpreted as being consistent with Sanders and Neville (2003a,b) provided that trials were systematically time-locked with word onsets). Namely, trials with mismatching auditory and visual stimuli yielded larger N400 amplitudes than trials with matching stimuli, which is consistent with interpretations of the N400 as “an electrophysiological marker of processing in a distributed semantic memory system” (Kutas and Federmeier, 2011).

Interestingly, a paradigm using similar stimuli with a different task yielded slightly different results. In the audiovisual integration task used in Hu et al. (2012), participants were instructed to focus on the visual stimuli and determine to which semantic category the words belonged (animals or colors), irrespective of the auditory stimuli. This version of the task also consistently yielded anterior peaks in the N100 and (late) N200 time-windows, but the relatedness between auditory and visual stimuli only affected the amplitude of the N200 peak, with matching auditory and visual pairs yielding smaller N200 amplitudes than mismatching pairs; (the N100 peak amplitude remained stable across conditions). Interestingly, an intermediate amplitude level was observed for stimulus pairs in which the visual did not match the auditory stimulus exactly, but consisted of a word from the same semantic category (another color or animal than the one heard).

Given that the audiovisual integration literature did not include L2 listeners, it can only directly support predictions regarding the audiovisual integration aspect of the present task. Namely, one might expect to observe peaks in the late N200 and N400 time-windows, the amplitude of which should vary as a function of the relationship between the auditory and the visual stimuli. That is, matching auditory and visual stimuli should yield smaller amplitude peaks in these two time-windows than mismatching stimuli pairs. Nonetheless, one can still make predictions with regard to how bilinguals might respond to the audiovisual integration aspect of the task. For instance, if L2 participants have difficulty adapting their use of L2-specific segmentation, one might expect to observe a smaller difference between matching and mismatching picture conditions caused by L2 participants' inaccurate segmentation of the ambiguous syllable strings. In other words, if the syllable strings are not segmented in a native-like (correct) manner, then the visual stimuli selected to match the auditory stimuli will not match the listener's faulty segmentation, effectively yielding ERPs comparable to the voluntarily mismatching stimuli. Conversely, if L2 participants were to present with ERPs similar to those of native listeners, then it would suggest that they segmented the auditory stimuli in a native-like manner.

## Method

### Participants

Seventy-four English-French bilingual listeners were recruited from the greater Montreal area, but 13 participants had to be removed from the dataset due to technical problems during the EEG recording, limited number of usable trials or missing information in the language history questionnaire. Data from 61 English-French bilingual adults (ranging between 18 and 36 years of age, mean 23.6 years, 16 males) were included in the analyses. Twenty-three participants reported French as their L1, 24 participants reported English as their L1, and 14 reported having been exposed to both languages from birth (hereafter referred to as “simultaneous” bilinguals). Of note, in the present paper, the terms “French-L1” and “English-L1” refer to participants who learned these languages (French or English) as an L1 and learned their L2 only later in life. Simultaneous bilinguals are not included in these categories even if they are native listeners of both languages. All participants spoke North American varieties of English and French. Written informed consent was obtained from every participant, and the research protocol was approved by the Institutional Review Board of McGill University's Faculty of Medicine & Health Sciences.

Information regarding language history and self-rated proficiency was collected through a questionnaire adapted from the *Language history questionnaire* (LHQ 2.0, Li et al., 2013) and all participants were right-handed according to the Edinburgh Handedness Inventory (Oldfield, 1971). Participants did not report any perceptual, speech, or learning impairments, as established during a pre-screening phone interview conducted to determine participants' eligibility, and presented normal or corrected-to-normal vision. All participants also presented normal hearing as assessed by a pure-tone average (PTA) hearing screening (thresholds <25 dB at 500, 1000, 2000, and 4000 Hz). Table 1 summarizes age of acquisition and language proficiency measures for the 61 participants included in the analyses.

**Table 1:** Age of acquisition and language proficiency measures of participants (self-reported and objective).

	**French L1**				**Simultaneous**				**English L1**			
	***M***	***SD***	***Min***	***Max***	***M***	***SD***	***Min***	***Max***	***M***	***SD***	***Min***	***Max***
Age of first L2 exposure	6.61	2.67	2	11	0	0	0	0	5.52	3.06	2	15
Self-reported proficiency^*^:												
French	6.59	0.45	5.50	7	6.00	1.06	4	7	5.54	0.81	4.50	7
English	5.88	1.05	4	7	6.50	0.62	5.50	7	6.93	0.17	6.50	7
Verbal fluency (total)												
French	50.22	11.14	25	70	48.21	12.12	26	69	38.75	9.72	22	60
English	49.35	11.79	28	69	63.93	14.76	45	103	63.75	11.56	39	89
Relative language dominance index^**^	1.01	0.28	0.56	1.82	1.38	0.40	1.08	2.65	1.72	0.43	1.08	2.47

* *Out of 7, where 1 = very poor and 7 = native-like*.

** *Total number of English words produced during verbal fluency task divided by total number of French words produced*.

Participants' relative language dominance was estimated by comparing the scores of verbal fluency tasks performed in English and in French. In the verbal fluency task, participants had 1 minute to name items corresponding to specific semantic or orthographic categories (e.g., “animals” or “words starting with the letter P”). The task included one semantic category and three different orthographic categories per language (“animals” and the letters F, A, and S in English; “fruits” and the letters P, F, and L in French). Participants performed the task first in their L1, then in their L2. Simultaneous bilinguals first performed the task in the language in which they felt most comfortable, and then in the other. The relative language dominance index was computed by dividing the total number of English words produced by participants across all conditions by the total number of French words produced across all conditions.

Of note, using a *relative* language dominance index presents the advantage of circumventing task-related effects by comparing each individual's performance across languages and incorporating the two scores into one continuous numerical value. Using a fixed formula where the English score is divided by the French score also means that the relative dominance index can be interpreted independently from the speaker's L1 (Birdsong, 2015). In the present sample, 47 participants obtained a relative language dominance index above one, indicating that they performed better in the English version of the task, suggesting that they are more proficient in English than in French, irrespective of their L1. Fourteen participants had a relative language dominance index below one, suggesting that they are more proficient in French than in English, regardless of their L1 (Birdsong, 2015; Treffers-Daller and Korybski, 2015). Such numbers suggest that most of our participants are dominant in English, which might be expected given that recruitment and testing was done in an anglophone institution (McGill University). On the other hand, this apparent language dominance imbalance might also be caused by the different letters and categories used in the English and French versions of the verbal fluency task. Research has suggested that speakers tend to produce more tokens overall in the “animals” category than in the “fruits” category (Moreno-Martínez et al., 2017; Gabrić and Vandek, 2020). Thus, the apparent language proficiency imbalance in favor of English might be caused by our use of an “easier” category in the testing of English proficiency. Regardless, this potential imbalance in difficulty of the semantic categories across languages would only affect the value at which participants would be considered balanced in terms of proficiency, not the overall interpretation of the relative language dominance index. Thus, the relative language dominance index has the advantage of providing scores on a continuous scale instead of assigning participants to categories (English dominant, French dominant, balanced), allowing the comparison of participants within a given sample.

### Stimuli

#### Overall Design

Given the constraints on the number of ambiguous word pairs and the structure of the sentences required for the present task, a limited set of stimuli could be used. Stimuli included recordings of 80 sentence pairs (40 pairs per language), each pair matched with 3 illustrations bearing different relationships with the auditory content (total of 240 illustrations). Each sentence of a pair was presented once with each of the three illustrations, for a total of 480 trials (240 per language). The following section details how the sentence recordings and illustrations were selected, controlled, and presented.

#### Sentence Content

Auditory stimuli consisted of recordings of the sentence pairs used in Gilbert et al. (2019). These sentences involve two-syllable strings that represent either one bisyllabic word or two monosyllabic words (e.g., in English, [kiwi] can be interpreted as “*kiwi*” or “*key we*”; in French, [ɔ ᖉ lɔʒ] can be interpreted as “*horloge*” —*Eng. clock*—or “*or loge*”—as in “*le vendeur d'or loge à*…” *Eng. the gold salesman is lodged at*). Within each pair, sentences were identical until the word(s) representing the two syllables of interest, and the grammatical content was controlled to the extent possible (see Gilbert et al., 2019 for details).

Given that word frequency has been found to influence the speed of lexical access (Segui et al., 1982) and that shorter words tend to have higher word frequencies (Zipf, 1935, 1949; Strauss et al., 2007), analyses were conducted to determine if the two conditions (one bisyllabic word or two monosyllabic words) involved lexical items with comparable frequencies. Subtitle-based lexical frequency for the bisyllabic words (“*kiwi*,” “*horloge*”) and the first word of the two monosyllabic words in the monosyllabic word condition (“*key*,” “*or*”) were extracted from *The English Lexicon Project* (Balota et al., 2007) and *Lexique 3* (New et al., 2011). Paired-sample *t*-tests were conducted for word frequencies from each language separately and revealed no significant differences between conditions for French trials (one word: *M* = 37.06, *SD* = 77.59, two word: *M* = 71.98, *SD* = 162.71, comparison: *t* = 1.227, *p* = 0.227), but revealed a significant difference between conditions for English trials (one word: *M* = 22.03, *SD* = 83.39, two word: *M* = 171.24, *SD* = 371.08, comparison: *t* = 2.433, *p* = 0.02). Upon inspection of the raw data, it appeared that the difference between English conditions might be caused by the presence of the highly frequent words “*man*” and “*two*” (present twice) in the two-word condition. When removing scores for these three sentence pairs from the dataset, the *t*-test becomes only marginally significant (one word: *M* = 22.03, *SD* = 83.39, two word: *M* = 77.60, *SD* = 133.28, comparison: *t* = 2.007, *p* = 0.053). Given these results, lexical frequency was included as a factor in all statistical models involving trial level behavioral data in an effort to control for its potential effect on the observed data patterns.

#### Stimuli Recording and Acoustic Characteristics

Sentences were recorded by a young adult simultaneous bilingual speaker (female), who had learned both English and French from birth. The speaker read each sentence three times in random order, to avoid over-emphasis on the difference between items of the pair (difference between *kiwi* and *key we*). The speaker was instructed to produce the sentences neutrally without inserting pauses, as if simply stating a fact. The speaker also took a breath between each sentence to ensure a complete reset of prosody. English and French sentences were recorded during two different recording sessions where the experimenter and speaker interacted in the to-be-recorded language in order to avoid code-switching effects. The recording was performed in a sound-attenuated booth, using a Shure (SM58) microphone and a Marantz PMD-670 digital recorder. Sentences were spliced into individual .*wav* files using *Goldwave* software (version 6.10), keeping 100 ms before the onset and after the offset of each sentence. Final recording selection was based on overall sound quality and absence of pauses or other disfluencies.

To ensure that the selected rendition of the sentences sounded natural, a short validation task was performed by 20 monolingual native listeners (10 per language). Participants listened to the sentences, in random order, and rated them from (1) *The sentence sounds perfectly natural; it is produced just like a native speaker would produce it*. to (4) *The sentence does not sound quite natural; not like we would expect from a native speaker*. An even number of levels was used to force participants to assign either a positive or negative rating to each sentence, avoiding midpoint ambiguous responses. Of note, 12 filler recordings were created by cross-splicing sentence parts across conditions to ensure the presence of clearly non-native-like productions. A total of 6 sentences (two French sentences and 4 English sentences) from different sentence pairs were rated at the less natural/native-like end of the continuum (3 or 4) by a majority of listeners (more than five out of 10) while all filler recordings received less natural/native-like ratings. Of note, removing these sentence pairs from the behavioral analyses had no significant impact on the observed pattern of results; they were therefore kept in the sample.

The selected recordings were also manually annotated by a trained bilingual coder in Praat (Boersma, 2001) following the method used in Gilbert et al. (2019). Acoustic information about each syllable of interest was extracted using custom scripts developed onsite. Offset latency of the second syllable of interest was also extracted to time illustration presentation. To quantify how these acoustic parameters vary across condition and to facilitate statistical comparisons, ratios were computed for each variable in each condition (e.g., in English [kiwi], Syl 1 = [ki] and Syl 2 = [wi]; Syl 2 score divided by the Syl 1 score; see Table 2). For instance, a duration ratio above one means that the second syllable of the ambiguous region is on average longer than the first syllable. Conversely, a duration ratio below one means that the second syllable is on average shorter than the first.

**Table 2:** Acoustic properties of first syllables (Syl 1) and second syllables (Syl 2) of the ambiguous region, and statistical comparison across conditions.

		**One word condition**						**Two word condition**						**Condition comparison of**			
														**Syl 2/Syl 1 ratios**			
		**Syl 1 ([ki])**		**Syl 2 ([wi])**		**Syl 2/Syl 1**		**Syl 1 ([ki])**		**Syl 2 ([wi])**		**Syl 2/Syl 1**					
		**M**	***SD***	**M**	***SD***	**M**	***SD***	**M**	***SD***	**M**	***SD***	**M**	***SD***	***b***	***SE***	***t***	***p***
English trials	Mean F0 (Hz)	217	*19*	220	*20*	1.02	*0.10*	215	*20*	211	*16*	0.99	*0.08*	−0.033	0.016	-2.112	0.041
	Mean duration (ms)	222	*61*	239	*70*	1.17	*0.50*	299	*82*	254	*96*	0.96	*0.54*	−0.207	0.065	-3.176	0.003
	Max amplitude (dB)	81	*2*	80	*4*	0.98	*0.04*	81	*2*	81	*2*	1.00	*0.03*	0.014	0.005	2.487	0.017
French trials	Mean F0 (Hz)	215	*13*	233	*16*	1.09	*0.1*	226	*15*	224	*20*	0.99	*0.09*	−0.095	0.017	-5.564	< 0.00001
	Mean duration (ms)	201	*49*	281	*52*	1.49	*0.48*	289	*58*	273	*75*	0.97	*0.28*	−0.517	0.062	-8.345	< 0.00001
	Max amplitude (dB)	81	*2*	82	*2*	1.01	*0.04*	82	*2*	81	*3*	0.99	*0.03*	−0.018	0.007	-2.747	0.009

Linear mixed effects regression models were used to determine if the different conditions were associated with different prosodic realizations (one word *kiwi* vs. two word *key we*). These models tested each language and each acoustic parameter separately and included a random intercept for sentence pair, to test the effect of condition over and above sentence pair specific differences. As reported in Table 2, the models yielded significant effects of condition for each acoustic parameter in each language, demonstrating that the stimuli in the two conditions were acoustically distinct.

#### Picture Selection

Three illustrations were selected for each sentence pair representing either the first monosyllabic word of the two word condition (Engl. “*key*” of “*key we*,” Fr. “*or*” of “*or loge*”), the bisyllabic word of the one word condition (Engl. “*kiwi*,” Fr. “*horloge*”) or something completely unrelated to either condition (total 240 illustrations, 120 per language). The unrelated illustrations did not match any of the words used in the sentence pair with which they were matched and the onset of their usual label did not present acoustic similarities with the syllables of interest or the usual labels for the related pictures. Most illustrations (86 in French and 80 in English) were pictures representing everyday items (object in color over a white background) selected from the *Bank of Standardized Stimuli* (BOSS, Brodeur et al., 2014). Others were collected through web searches and included more complex scenes. The link between the illustration and the word it represents was validated by 26 native speakers (13 in each language). Raters were asked to evaluate the picture/word relationship using a 7-point scale from 1) “*Perfectly illustrates (could be used as illustration in a dictionary)*” to 7) “*Does not illustrate (no link whatsoever)*.” All English pictures and all but two French pictures were rated on the positive side of the scale by a majority of raters (at least seven participants out of thirteen). The two French pictures that received lower ratings relate to concepts that are difficult to illustrate (“*voix*” and “*taux*,” which translate to “*voice*” and “*rate*”). Of note, removing these two sentence pairs from the behavioral analyses of French trials had no significant impact on the observed pattern of results. The sentence pairs were therefore kept in the sample and part of the variability they might cause should be accounted for by the use of random slopes for picture conditions in the random effects structure.

### Procedure

Data collection was done over two sessions on different days. On their first visit, participants performed the verbal fluency tasks, completed the language history questionnaire, and participated in another experiment running at the lab at that time (a bilingual speech production task performed in both English and French, not reported here). On their second visit, participants underwent the audiometric screening, completed the present EEG task as well as other EEG tasks running at the lab at the time (a bilingual speech processing task performed in both English and French, not reported here).

In the present EEG task, trials were grouped into two blocks of stimuli per language (4 blocks in total). Each block contained 120 trials with an equal number of sentences of each condition (one bisyllabic word or two monosyllabic words). Sentence order within blocks was fixed and pseudo-randomized to minimize the risk of participants noticing the existence of sentence pairs. Participants first performed the two blocks of their L1 (block order within language balanced across listeners) and then the two blocks of their L2. Simultaneous bilinguals were asked to determine in which language they felt most comfortable and performed the task first in that language and then in their other native language. Language order was fixed among sequential bilinguals, taking for granted that most speakers would be dominant in their L1, creating comparable testing conditions for sequential and simultaneous bilinguals. Participants were offered breaks between blocks.

Each trial began with a fixation cross at the center of the screen for 500 ms before the sentence started playing. The fixation cross remained onscreen until the offset of the first syllable of interest. Then the illustration replaced the fixation cross on the screen and remained visible until the end of the trial. Participants were instructed to determine, as quickly as possible, if the illustration was related to the sentence using a key press. Half the participants responded “related” with the right hand and “unrelated” with the left hand; the remaining participants used the reverse correspondence of keys. Responses were scored as accurate when participant responded “related” to the image corresponding to the heard sentence or “unrelated” to the image corresponding to the other sentence of the pair. Participants performed a block of five practice trials before the first block of each language to become familiar with the task and avoid contamination of the data by language switching effects. Each sentence of a pair was presented once with each of its three illustrations leading to six sentence/picture conditions as illustrated in Table 3.

**Table 3:** Schematic representation of sentence and picture condition combinations in English and French trials.

		**Picture condition**		
**Sentence condition**		**“Exact match”**	**“Match other”**	**Completely unrelated**
English	One word	*If you would like a kiwi, we …*	*If you would like a kiwi, we …*	*If you would like a kiwi, we …*
		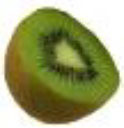	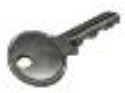	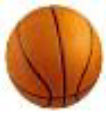
	Two word	*If you would like a key, we …*	*If you would like a key, we …*	*If you would like a key, we …*
		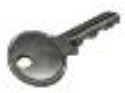	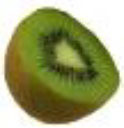	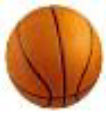
French	One word	*Le vendeur d'horloge …*	*Le vendeur d'horloge …*	*Le vendeur d'horloge …*
		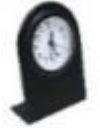	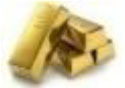	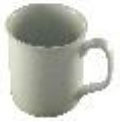
	Two word	*Le vendeur d'or loge …*	*Le vendeur d'or loge …*	*Le vendeur d'or loge …*
		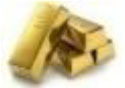	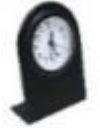	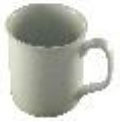

Auditory stimuli were presented at 66 dBA *via* insert earphones (EARTONE 3A) and participants sat about 60 cm from the computer screen. The EEG session lasted ~4 h including preparation time (~30 mins), breaks between blocks (~10 mins) and other tasks (not reported here).

### EEG Recording and ERP Analyses

#### EEG Recording

EEG was continuously recorded using 64 Ag/AgCl active electrodes positioned according to the enhanced 10/20 system (2.048 kHz sampling rate, 24-bit resolution, 0.1 to 1000 Hz bandwith, BioSemi Active-Two system). EEG electrodes were online-referenced to the right earlobe. Vertical eye movements were recorded using bipolar electrodes placed above and below the right eye while horizontal eye movements were recorded using electrodes placed at the outer canthi of the eyes.

#### EEG Analyses

##### Data Preprocessing

Data preprocessing and ERP quantification was done using BrainVision Analyser2 (version 2.1, Brain Products GmbH, Germany). EEG signals were filtered offline (bandpass between 0.1 to 30Hz) and then re-referenced to the average of the left and right mastoids. Eye-movement artifacts generated by blinks and saccades were identified and removed using the *Ocular Correction ICA* transformation implemented in BrainVision Analyser2. The continuous EEG was segmented into epochs time-locked to the onset of the illustration presentation (500 ms before to 1000 ms after) and only trials for which the participants provided the correct response were selected for analysis. Participants provided on average 31 correct responses per condition pair across both languages (out of 40 trials per condition pair in each language; English *SD* = 6.24, French *SD* = 6.93). Rejection of correct response segments containing artifacts was done using manually verified automatic artifact rejection routines. Participants presented on average 22.67 artifact-free correct response French trials (*SD* = 7.22) and 22.54 artifact-free correct response English trials (*SD* = 7.26) per condition pair. In other words, the limited number of trials included in the final EEG analyses can be explained by the application of two selection criteria: incorrect responses leading to the rejection of, on average, 9 trials, and signal artifacts leading to the rejection of, on average, 8 trials per condition pair. Artifact-free correct-response epochs were baseline corrected to the 200 ms time-window preceding presentation of the illustration. Of note, although one usually tries to baseline correct ERPs according to a time-window containing no stimulus induced activity, it would not be feasible in the present paradigm given the variability in picture onset latencies and sentence structure across sentence pairs. By using a fixed time-window prior to illustration presentation, one can at least ensure that the content of the baseline is comparable across items of the same sentence pair (i.e., the baseline includes part of the [ki] in both *key we* and *kiwi*). Epochs were then averaged for each condition and participants separately.

##### ERP Quantification

Grand averages across all participants were computed for each language taking into account sentence and picture conditions. Within each language, the two-word condition with a matching picture was used as a reference for peak detection since it is the condition with the clearest link between sentence and picture (in English, the participant hears “key” immediately followed by the picture of a key). Automatic peak detection routines applied within the traditional N1 and N2 time-windows located their peaks at 105 ms and 230 ms respectively in the present dataset. Therefore, mean N1 and N2 amplitude were extracted using a 100 ms time-window around the grand average peak for each participant, condition, and electrode (N1: from 55 to 155 ms, N2: from 180 to 280 ms). Given that N4 effects are not always associated with clear peaks, mean amplitudes were simply extracted over a 100ms time-window centered around 400ms (from 350 to 450 ms).

##### Localization of Regions of Interest (ROIs)

A series of linear mixed effect models were used to guide the selection of a region of interest in which to investigate the effect of individual differences between participants (see *Statistical design section* for software details). The same analysis scheme was applied to N1, N2 and N4 mean amplitudes in both languages. A first model was used to locate the components along the anterior-posterior dimension by testing the effect of electrode location on the midline (from Afz to POz) along with task-related effects (sentence and picture conditions) on participants' mean ERP amplitudes (N1, N2 or N4). FCz was selected as the reference location based on visual inspection of topographic maps of the English reference condition. These models also included random effects for participants (intercepts only). None of the models revealed a significant interaction between anterior-posterior position and condition, but they all revealed significant main effects of anteriority, with frontal electrodes yielding more negative ERPs than posterior electrodes. These results demonstrated that the N1, N2 and N4 components were maximally observable at anterior sites.

A second model was then used to locate each components (N1, N2 and N4) in the laterality dimension. To do so, two regions of interest were created by averaging mean amplitudes over a left frontal ROI (Af3, F1, F3, F5, FC1, FC3, FC5) and a right frontal ROI (Af4, F2, F4, F6, FC2, FC4, FC6) for each participant and condition (models also included random intercepts for participants). None of the models revealed a significant interaction between laterality and conditions, and the main effect of laterality never reached significance, indicating that the observed components were equally observable at left and right electrodes. In order to determine if participants' L1 had an impact on the laterality of the effects, participant L1 was added to the models as an interaction factor. The models did not yield significant interactions between L1 and laterality, indicating that the localization of the components did not vary as a function of participants' L1. Therefore, the same ROI has been used to investigate individual differences in ERP amplitudes across listener groups.

Given the absence of a laterality effect, the analyses investigating the impact of individual differences on N1, N2 and N4 amplitudes were performed on a frontal ROI (including the previously mentioned left and right ROIs and the corresponding midline electrodes) from participant grand averages computed as a function of sentence and picture condition, as well as language of the trial.

### Statistical Design

Behavioral and ERP data were analyzed using a series of linear mixed effects (LME) models on English and French trials separately. Of note, trials with completely unrelated pictures were not included in the present analyses, as they do not provide information regarding the correct processing of the ambiguous syllable strings. A first model was designed to determine the effects of sentence condition (two-level deviation or sum coded categorical variable, comparing each condition to the overall mean) and picture condition (two-level treatment or “dummy” coded categorical variable: “exact match” or “match other” sentence of the pair, reference level set to “exact match” condition, see Schad et al. (2020), for a discussion of a priori contrasts determination). The second and third models included interactions between conditions (sentence and picture) and language experience variables such as listeners' L1 [as a three-level treatment coded categorical variable; English-L1, French-L1, and simultaneous bilingual; reference level set to L1 speakers of the language being analyzed, thus comparing the two other listener groups to L1 speakers of that language; UCLA: Statistical Consulting Group (2011)] and the listeners' relative language dominance index (as a scaled continuous variable based on the relative verbal fluency scores). Given the potential collinearity between L1 and language dominance, the two variables were tested separately (Tabachnick and Fidell, 2007; Baayen, 2008; Baayen et al., 2008). Of note, trial level behavioral data were analyzed using binomial models and included word frequency of the syllables of interest (log transformed) to control for its potential effect on the response accuracy and reaction times.

Furthermore, random slope adjustments were included in the random structure of the models only where it was “justified by the design” (Barr et al., 2013; see also Bates et al., 2015, for an argument in favor of “Parsimonious mixed models”). Thus, the random structure of the behavioral models took into account participants (intercept only) and items (i.e., sentence pairs), with intercept and slope adjustments for picture conditions; i.e., [Accuracy Score ~ Sentence Condition ^*^ Picture Condition ^*^ scale(Relative language dominance index) + (1 | Participant) + (1 + Picture Condition | Sentence Pair)]. Random slope adjustments for picture conditions were included to compensate for the fact that we could not completely control the strength of the relationship between pictures and sentences. Including such a random slope adjustment allowed the observation of condition effects over and above the specifics of each sentence pair. On the other hand, the impact of participant-level variables should not vary across speakers, and therefore no slope adjustments were applied to the random effect of participants. Of note, the random structure of ERP models took into account only participants (intercept) since the models did not include trial level data (only means per conditions for each participant). When models failed to converge, variables were removed one by one, until convergence, beginning with random slopes on items and then removing higher order interactions. Of note, we tried using the interaction between sentence and picture conditions as random slope adjustments on items but the majority of these models failed to converge.

LME models were implemented in RStudio version 3.2.4 (R Development Core Team, 2010), using the lme4 library, version 1.1-7 (Bates et al., 2014). Estimates of p-values were obtained using the lmerTest package version 2.0-29 (Kuznetsova et al., 2015), while estimates of model fit were obtained using the MuMIn package version 1.43.17 (Barton, 2020, providing R squared scores) and the Stats package version 4.0.3 (Sakamoto et al., 1986, providing AIC and BIC scores). Plots were generated using ggplot2 (version 2.1.0, Wickham, 2009) and Excel as implemented in Office 365. Description of the significant results are reported in the *Results section* and a summary of significant results per dependent variable is provided in Table 4 (*Discussion section*). Complete model outputs are available in Supplementary Materials (Meteyard and Davies, 2020).

**Table 4:** Summary of significant results.

**Language**	**Category of effects**	**ERPs**			**Behavioral**	
		**N1**	**N2**	**N4**	**Accuracy**	**Reaction times**
French trials	Experimental conditions	NA	Sentence by Picture	Sentence^*^Picture^*^	Sentence by Picture	Sentence^*^Picture^*^
			*2word:exact match^1^ < all other conditions*	*1word > 2word Exact match^1^ < match other^2^*	*2word:exact match^1^ < all other conditions*	*1word < 2word Exact match^1^ < match other^2^*
	Language experience variables	L1	NA	Sentence by Relative language dominance	L1^*^ Relative language dominance^*^	L1^*^
		*Engl.-L1 < native Fre*.		*Fre. dom: 1word > 2wordEngl. dom: 1word ~ 2word*	*Engl.-L1 < native Fre. Engl. dom < Fre. dom*	*Engl.-L1 > native Fre*.
English trials	Experimental conditions	Sentence by Picture	Sentence by Picture	Sentence^*^Picture^*^	Sentence by Picture	Sentence^*^Picture^*^
		*2word:exact match^1^ < all other conditions*	*2word:exact match^1^ < all other conditions*	*2word < 1word Exact match^1^ < match other^2^*	*2word:match other^2^ < all other conditions*	*1word < 2word Exact match^1^ < match other^2^*
	Language experience variables	NA	NA	NA	Picture by L1	Picture by L1
					*Native Fre.:match other^2^ < all other conditions*	*Larger diff between pic conditions for simulaneous*

* *Main effects only*.

1 *“Exact match” = picture that represents the heard sentence*.

2 *“Match other” = picture that represents the other sentence of the pair*.

## Results

### Event-Related Potentials

#### French Trials

**Figure 1:**
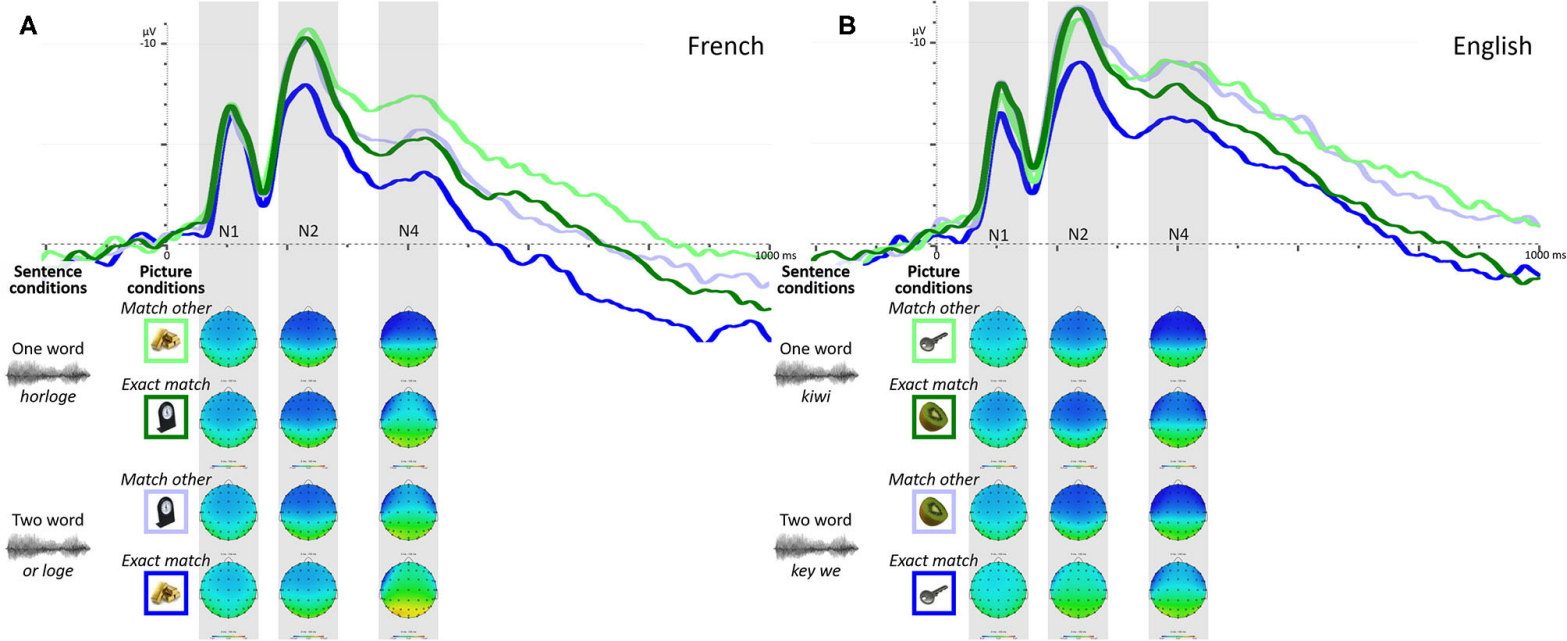
Grand average ERPs over frontal ROI to each sentence and picture condition combination for French **(A)** and English **(B)** trials, along with topographic representation over time-windows used for ERP quantification (shadowed boxes). Green waveforms represent ERPs to the one word condition while blue waveforms represent the two word condition; darker colors represent the “exact match” picture condition while the lighter colors represent the “match other” condition.

One participant was removed from the ERP analyses because of too few artifact-free correct-response trials. Figure 1 presents the grand average ERPs for each sentence and picture condition combination for French trials for the remaining 60 participants. The waveform presents two clear negative deflections peaking around 100ms and 250ms from picture onset (hereafter N1 and N2), although only the N2 shows signs of being affected by the experimental conditions. A third less-defined peak is also observable in the typical N400 time-window (hereafter N4), the amplitude of which also seems to be influenced by the experimental conditions.

##### N1 Amplitude

As expected from Figure 1, the LME model testing the effect of condition on N1 amplitudes yielded no significant main effect of sentence or picture condition, nor an interaction between factors [*b* < 0.3685, *SE* > 0.1913, *t* < −1.362, *p* > 0.1740]. Adding participants' L1 as an interaction factor to the model yielded a significant main effect of L1 for native English listeners [*b* = 1.6149, *SE* = 0.6962, *t* = 2.320, *p* = 0.0233], who presented with lower N1 amplitudes than native French listeners, but not for simultaneous bilinguals [*b* = 0.8468, *SE* = 0.7509, *t* = 1.128, *p* = 0.2633]. All (higher- or lower- level) interactions involving L1 failed to reach significance [*b* < 0.7226, *SE* > 0.4482, *t* < −1.400, *p* > 0.1623]. Replacing L1 by the relative language dominance index revealed no significant effect of the language dominance index as a main effect or as part of any interaction [*b* < 0.4737, *SE* > 0.1918, *t* < 1.574, *p* > 0.1200].

##### N2 Amplitude

The LME model testing the effect of condition on N2 amplitudes revealed a significant interaction between sentence and picture conditions [*b* = −1.5952, *SE* = 0.6457, *t* = −2.471, *p* = 0.0144] with picture condition (“exact match” vs. “match other”) being associated with greater N2 amplitude differences in the two word condition (*or loge*) than the one word condition (*horloge*). This result is reflected in the lower N2 amplitude observed in Figure 1 for trials where participants heard the two word sentence condition (*or loge*) paired with a matching picture (picture of a gold bar—Fr. *or*).

Simply adding participants' L1 as an interaction factor in the model revealed no significant effect of L1 as a main effect or as part of interactions [*b* < 0.8597, *SE* > 0.7650, *t* < −1.042, *p* > 0.2990]. Of note, the interaction between sentence and picture conditions failed to reach significance in this model, probably due to overfitting.

The model including the relative language dominance index instead of L1 yielded a significant interaction between sentence and picture conditions [*b* = −1.5952, *SE* = 0.6475, *t* = −2.464, *p* = 0.0147], while the effect of relative language dominance failed to reach significance as a main effect or as part of any (higher- or lower- level) interaction [*b* < −0.3509, *SE* > 0.3244, *t* < −0.541, *p* > 0.5893].

##### N4 Amplitude

The model testing the effect of sentence and picture conditions on N4 amplitudes yielded no significant interaction between factors [*b* = −0.2687, *SE* = 0.8211, *t* = −0.327, *p* = 0.7439], but revealed two main effects, as expected from Figure 1. A main effect of sentence condition was observed [*b* = 1.7051, *SE* = 0.5806, *t* = 2.937, *p* = 0.0038] where the two word condition (*or loge*) was associated with lower N4 amplitudes than the one word condition (*horloge*); a main effect of picture condition also emerged [*b* = −2.0944, *SE* = −0.4105 *t* = −5.102, *p* < 0.0001], where the “exact match” picture condition was associated with lower N4 amplitudes than the “match other” picture condition (seeing the picture associated with the other sentence condition; for example, seeing the picture of a gold bar—Fr. *or*—when hearing the *horloge* sentence).

Adding participants' L1 as an interaction factor to the model yielded no significant effect of L1 as a main effect or as part of any interaction [*b* < 0.7394, *SE* > 0.9728, *t* < −0.550, *p* > 0.5834]. The main effect of picture condition remained significant [*b* = −1.9975, *SE* = 0.6720, *t* = −2.972, *p* = 0.0034], but the main effect of sentence condition and the interaction between conditions failed to reach significance [*b* < 1.6737, *SE* > 0.9504, *t* < 1.761, *p* > 0.0800].

In turn, adding the relative language dominance index instead of L1 revealed a significant interaction between sentence condition and relative language dominance [*b* = −1.2989, *SE* = 0.5779, *t* = −2.248, *p* = 0.0259] with participants at the French dominant end of the spectrum presenting with different N4 amplitudes for the two sentence conditions (one word condition—*horloge*—presenting with larger N4s than the two word condition—*or loge*), while participants at the English dominant end of the spectrum showed similar N4 amplitudes in response to both sentence condition.

#### English Trials

Three participants were removed from the analyses because of too few artifact-free correct-response trials. Figure 1 presents the grand average ERPs for each sentence and picture condition combination for English trials for the remaining 58 participants. As observed in French trials, the waveform presents two clear negative deflections (N1 and N2) as well as a third less defined peak (N4). But unlike French trials, here the amplitude of all three peaks shows signs of being affected by the experimental conditions.

##### N1 Amplitude

The LME model testing the effect of experimental conditions on N1 amplitudes revealed a significant interaction between sentence and picture conditions [*b* = −1.1999, *SE* = 0.3994, *t* = −3.004, *p* = 0.0028], with picture condition being associated with larger N1 amplitude differences in the two word condition (*key we*) than the one word condition (*kiwi*). This result is reflected in the lower N1 amplitude observed in Figure 1 for trials where participants heard the two word sentence condition (*key we*) and were presented with a matching picture (picture of a key). The model also revealed a main effect of sentence condition [*b* = 1.0452, *SE* = 0.2816, *t* = 3.711, *p* = 0.0002], but not of picture condition [*b* = −0.2022, *SE* = 0.1997, *t* = −1.012, *p* = 0.3119].

Adding participants' L1 as an interaction factor to the model yielded no significant effect of L1 as a main effect or as part of any lower- or higher-level interaction [*b* < 1.0679, *SE* > 0.5055, *t* < −1.505, *p* > 0.1368], while the significant interaction between sentence and picture conditions was maintained [*b* = −1.5730, *SE* = 0.6648, *t* = −2.366, *p* = 0.0185]. The model including the relative language dominance index instead of L1 also yielded no significant effect of language dominance as a main effect or as part of any interaction [*b* < −0.3500, *SE* > 0.2000, *t* < 1.365, *p* > 0.1729], while the significant interaction between sentence and picture conditions was maintained [*b* = −1.2033, *SE* = 0.3994, *t* = −3.013, *p* = 0.0028].

##### N2 Amplitude

The LME model testing the effect of conditions on N2 amplitudes revealed a significant interaction between sentence and picture conditions [*b* = −2.5665, *SE* = 0.8066, *t* = −3.182, *p* < 0.0016] with picture condition (“exact match” vs. “match other”) associated with greater N2 amplitude differences in the two word condition (*key we*) than the one word condition (*kiwi*). As in French trials, this result is reflected in the lower N2 amplitude observed in Figure 1 for trials where participants heard the two word sentence condition (*key we*) and were presented with a matching picture (picture of a key).

As in the French trials, adding participants' L1 to the model yielded no significant interactions involving L1 as well as no main effect of L1 [*b* < −1.7652, *SE* > 0.9547, *t* < −1.275, *p* > 0.2065]. But unlike in French trials, the interaction between sentence and picture conditions remained significant in this model [*b* = −2.8871, *SE* = 1.3448, *t* = −2.147, *p* = 0.0324].

Replacing L1 by the relative language dominance index again yielded a significant interaction between sentence and picture conditions [*b* = −2.5770, *SE* = 0.8076, *t* = −3.191, *p* < 0.0015], but no significant effect of relative language dominance either as a main effect or as part of any lower- or higher-level interaction [*b* < 0.5471, *SE* > 0.4045, *t* < 1.353, *p* > 0.1770].

##### N4 Amplitude

The model testing the effect of sentence and picture conditions on N4 amplitudes yielded no significant interaction between factors [*b* = −0.9386, *SE* = 0.8472, *t* = −1.108, *p* = 0.2686], but revealed a main effect of sentence condition [*b* = 1.4036, *SE* = 0.5974, *t* = 2.350, *p* = 0.0193], with the two word condition (*key we*) being associated with lower N4 amplitudes than the one word condition (*kiwi*), and a main effect of picture condition [*b* = −1.8371, *SE* = 0.4236, *t* = −4.337, *p* < 0.0001], where the “exact match” picture condition was associated with lower N4 amplitudes than the “match other” picture condition (seeing the picture associated with the other sentence condition; for example, seeing the picture of a key when hearing the *kiwi* sentence).

Adding participants' L1 to the model yielded no main effect of L1 and no significant interactions involving L1 [*b* < −1.4094, *SE* >1.0003, *t* < −1.004, *p* > 0.3160]. Of note, the main effect of sentence condition failed to reach significance in this model, probably due to overfitting.

Replacing L1 by the relative language dominance index revealed no significant effects of relative language dominance as a main effect or as part of any interaction [*b* < 0.7194, *SE* > 0.4241, *t* < 1.696, *p* > 0.0906] while the main effect of experimental conditions remained significant [*b* > 1.4036, *SE* < 0.5970, *t* > 2.351, *p* < 0.0192].

### Behavioral Results

Trials with response latencies below 100 ms and above 2,000 ms (longer than the shortest sentence stimuli) were removed from the analyses, which led to the rejection of 1365 English trials (out of 10,240, or 13.3% of trials) and of 772 French trials (out of 10,159, or 7.6% of trials). The removed trials presented comparable accuracy rates, suggesting that their removal would not have significantly impacted the analyses of accuracy scores (removed trials: English = 73% correct, French = 77% correct; kept trials: English = 78% correct; French = 79% correct; see Supplementary Materials for a detailed breakdown of rejected trials as a function of sentence and picture conditions).

#### French Trials

**Figure 2:**
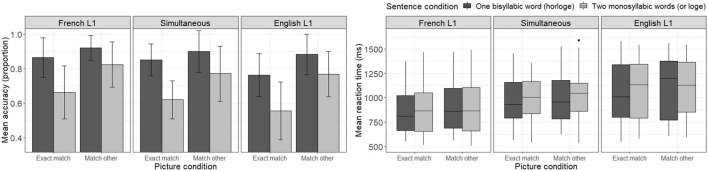
Mean accuracy **(left)** and reaction time (ms) to correct response **(right)** to French trials as a function of sentence and picture conditions, as well as participants' native language.

Figure 2 presents mean accuracy scores and reaction times to correct responses during French trials for each sentence and picture condition combination as a function of participants' native language. Looking at the accuracy panel of Figure 2, one can make four main observations. First, one notices that accuracy levels are in general higher in the one word condition (*horloge*—dark bars) than in the two word condition (*or loge*—light bars). Second, one notices that the “match other” picture condition (where participants see the picture matching the other sentence condition; e. g., seeing the picture of *gold bars*—Fr. *or*—when hearing the *horloge* condition—En. *clock*) seems to yield higher accuracy rates than the “exact match” condition within participant groups. Namely, participants seem to be better at rejecting the relationship between the heard sentence and the picture representing the other sentence of the pair (e.g., determining that the picture of gold bars—Fr. *or*—is not related to the one word condition—*horloge*) than at recognizing the link between the heard sentence and the matching picture (e.g., determining that the picture of a clock—Fr. *horloge*—is related to the one word auditory stimulus—*horloge*). Third, comparing listener groups, one notices that English-L1 listeners tend to have lower accuracy levels, particularly in the “exact match” picture condition, than other listener groups. And finally, no listener group reached a perfect accuracy level in any condition, not even French-L1 listeners. A less structured pattern of results is observed in the reaction time panel of Figure 2, where each subgroup of participants seems to present with a different pattern of reaction time distributions.

##### Response Accuracy

The LME model testing the effect of condition on accuracy scores across participants revealed a significant interaction between sentence and picture conditions [*b* = 0.3259, *SE* = 0.1284, *z* = 2.538, *p* = 0.0112], with picture type (“exact match” vs. “match other”) having a greater impact on accuracy in the two word condition (*or loge*) than the one word condition (*horloge*). This result is reflected in lower accuracy scores observed in Figure 2 for trials where participants heard the two word sentence condition (*or loge*) paired with a matching picture (picture of a gold bar—Fr. *or*).

The model including participants' L1 as an interaction factor failed to converge when including a random slope for picture condition. A simplified model without the random slope yielded no significant lower- or higher-level interactions involving participants' L1 [*b* < 0.2664, *SE* > 0.1409, *z* < 1.497, *p* > 0.1189], but revealed a significant main effect of L1 only for English-L1 participants [*b* = −0.7620, *SE* = 0.2384, *z* = −3.196, *p* = 0.0014] (not for simultaneous bilinguals [*b* = −0.2593, *SE* = 0.2776, *z* = −0.934, *p* = 0.3503]). Namely, only English-L1 participants presented with different (lower) accuracy scores compared to the French-L1 participants used as reference here. Of note, the previously observed significant interaction between sentence and picture conditions failed to reach significance in this model, probably due to overfitting.

The model including the relative language dominance index as an interaction factor instead of L1 also failed to converge when including a random slope for picture condition. A simplified model without the random slope yielded no significant interactions involving the relative language dominance index [*b* < 0.0903, *SE* > 0.0569, *z* < 1.222, *p* > 0.222], but revealed a significant main effect of relative language dominance [*b* = −0.4385, *SE* = 0.0921, *z* = −4.759, *p* < 0.0001], with higher relative language dominance indices (suggesting English dominance) being associated with lower accuracy scores in general. The interaction between experimental conditions also remained significant [*b* = 0.2564, *SE* = 1261, *z* = 2.034, *p* = 0.042].

##### Reaction Time to Accurate Responses

The LME model testing the effect of condition on reaction times to accurate responses across participants revealed significant main effects of sentence condition [*b* = 59.2200, *SE* = 9.9930, *t* = 5.926, *p* < 0.0001] and picture condition [*b* = 38.3800, *SE* = 14.0760, *t* = 2.727, *p* = 0.0098], while the interaction between factors failed to reach significance [*b* = −7.9320, *SE* = 12.8570, *t* = −0.617, *p* = 0.5373]. Namely, participants responded faster to the one word sentence condition (*horloge*) compared to the two word sentence condition (*or loge*), and faster to the “exact match” picture condition compared to the “match other” picture condition (when seeing a picture corresponding to the other sentence of the pair).

Adding participants' L1 as an interaction factor in the model yielded a significant main effect of L1 for English-L1 listeners [*b* = 199.9090, *SE* = 83.4530, *t* = 2.3950, *p* = 0.0198], who presented with slower reaction times than French-L1 listeners (used as reference). The main effect of L1 for simultaneous bilinguals failed to reach significance [*b* = 99.9060, *SE* = 96.8630, *t* = 1.031, *p* = 0.3066], but a marginally significant 3-way interaction was observed across sentence condition, picture condition and L1 for simultaneous bilinguals [*b* = 61.6400, *SE* = 32.7130, *t* = 1.884, *p* = 0.0596]. That is, within the “match other” picture condition, simultaneous bilinguals presented with larger reaction time differences across sentence conditions. All participants took longer to determine that the “match other” picture was not related to the two word sentence condition (seeing a clock—Fr. *horloge*—while hearing *le vendeur d'or loge*—En. *the gold salesman is lodging*) than to the one word sentence condition (seeing a gold bar—Fr. *or*—while hearing *le vendeur d'horloge*—En. *the clock salesman*), but simultaneous bilinguals presented with a larger reaction time difference across conditions compared to the French-L1 listeners used as reference. Contrary to previous results, the main effect of picture condition failed to reach significance in the present model [*b* = 28.2570, *SE* = 15.9480, *t* = 1.772, *p* = 0.0816], while the main effect of sentence condition was preserved [*b* = 56.847, *SE* = 14.7430, *t* = 3.856, *p* = 0.0001].

Replacing L1 by the relative language dominance index yielded significant main effects of sentence condition [*b* = 59.4931, *SE* = 10.0031, *t* = 5.947, *p* < 0.0001] and picture condition [*b* = 38.4823, *SE* = 14.0981, *t* = 2.730, *p* = 0.0089], but only a marginally significant effect of the relative language dominance index [*b* = 69.5671, *SE* = 35.0867, *t* = 1.983, *p* = 0.0520], with participants at the English dominant end of the spectrum presenting with longer reaction times overall than participants at the French dominant end of the spectrum. All lower- or higher- interactions involving relative language dominance failed to reach significance [*b* < 17.3636, *SE* > 6.3954, *t* < 1.365, *p* >0.1723].

#### English Trials

**Figure 3:**
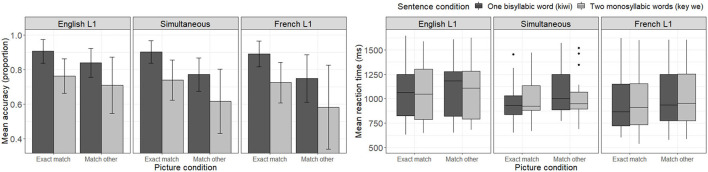
Mean accuracy **(left)** and reaction time (ms) to correct response **(right)** to English trials as a function of sentence and picture conditions, as well as participants' native language.

Figure 3 presents mean accuracy scores for each sentence and picture condition combination as a function of participants' L1. Four main observations may be made from visual inspection of the Figure. One first notices that, as in French trials, accuracy levels seem higher in the one word condition (*kiwi*—dark bar) than in the two word condition (*key we*—light bar). Second, unlike what was observed for French trials, in the English trials, the “exact match” picture condition seems to yield higher accuracy rates within speaker groups than the “match other” picture condition. Namely, participants seem to be better at recognizing the relationship between the heard sentence and the matching picture (e. g., determining that the picture of a kiwi is related to the one word condition—*kiwi*) than at rejecting the link between the heard sentence and the picture representing the other sentence of the pair (e. g., determining that the picture of a key is not related to the one word condition—*kiwi*). Third, comparing listener groups, one notices that English-L1 listeners seem to have higher accuracy levels than both other groups, particularly in the “match other” condition. And finally, as in French trials, no listener group reached a perfect accuracy level in any condition, not even English-L1 listeners. Turning our attention to the reaction time panel, one notices that, as in French trials, each subgroup of participants seems to present with a different pattern of reaction time distributions.

##### Response Accuracy

The LME model testing the effect of conditions on accuracy scores across participants revealed a significant interaction between sentence and picture conditions [*b* = 0.4587, *SE* = 0.1331, *z* = 3.446, *p* = 0.0006], with picture type (“exact match” vs. “match other”) having a greater impact on accuracy in the one word condition (*kiwi*) than the two word condition (*key we*). The model in which L1 was added as an interaction factor failed to converge. A simplified model without random slopes revealed a significant interaction between picture condition and participant L1 [French-L1: *b* = −04846, *SE* = 0.1454, *z* = −3.333, *p* = 0.0009; Simultaneous bilinguals: *b* = −0.3775, *SE* = 0.1655, *z* = −2.280, *p* = 0.0226]. Namely, the two subgroups of listeners showed lower accuracy on the “match other” picture conditions compared to English-L1 listeners, while their accuracy was comparable to English-L1 listeners on the “exact match” picture conditions. Of note, the interaction between sentence and picture conditions remained significant [*b* = 0.4673, *SE* = 0.2130, *z* = 2.194, *p* < 0.0282].

Like the previous model, the model including the relative language dominance index yielded a significant interaction between sentence and picture conditions [*b* = 0.5086, *SE* = 0.1257, *z* = 4.047, *p* < 0.0001], while the effect of the relative language dominance index failed to reach significance as a main effect or as part of any lower- or higher-level interaction [*b* < 0.0632, *SE* > 0.0606, *z* < 1.041, *p* > 0.2980].

##### Reaction Time to Accurate Responses

As in the French trials, the LME model testing the effect of condition on reaction time to accurate responses for English trials revealed significant main effects of sentence condition [*b* = 37.9353, *SE* = 11.2352, *t* = 3.376, *p* < 0.0007] and picture condition [*b* = 104.3690, *SE* = 11.5215, *t* = 9.059, *p* < 0.0001], while the interaction between factors failed to reach significance [*b* = −20.0453, *SE* = 14.3386, *t* = −1.398, *p* = 0.1622]. Participants responded faster to the one word sentence condition (*kiwi*) compared to the two word sentence condition (*key we*), and faster to the “exact match” picture condition compared to the “match other” picture condition (when seeing a picture corresponding to the other sentence of the pair).

Adding participants' L1 as an interaction factor in the model yielded no significant main effects of L1 [*b* < −120.5438, *SE* > 86.9087, *t* < −1.387, *p* > 0.1707], but revealed a significant interaction between picture condition and L1 for simultaneous bilinguals [*b* = 48.9032, *SE* = 18.3226, *t* = 2.669, *p* = 0.0076] only. That is, simultaneous bilinguals presented with larger reaction time differences across picture conditions compared the English-L1 listeners (used as reference). Thus, the “match other” picture condition seems to have slowed simultaneous bilinguals down more than English-L1 listeners, regardless of both groups being composed of native English speakers. All other interactions involving L1 failed to reach significance [*b* < −20.4658, *SE* > 16.0902, *t* < 1.210, *p* > 0.02263], but the significant main effects of sentence and picture condition were maintained (sentence condition: [*b* = 39.1899, *SE* = 16.3927, *t* = 2.391, *p* = 0.1707], picture condition [*b* = 85.8184, *SE* = 14.4055, *t* = 5.957, *p* < 0.0001]).

On the other hand, replacing L1 by the relative language dominance index yielded no significant effect of relative language dominance as a main effect or as part of any interaction [*b* < 35.3024, *SE* > 7.1081, *t* < 0.937, *p* > 0.3525]. Both main effects of condition remained significant (sentence condition: [*b* = 37.9031, *SE* = 11.2374, *t* = 3.373, *p* = 0.0007], picture condition: [*b* = 104.3427, *SE* = 11.5109, *t* = 9.065, *p* < 0.0001]).

## Discussion

The goals of the present study were to determine if English-French bilinguals are able to adapt their speech segmentation strategies to the specifics of their L2, if individual differences with language experience (as indexed by participants' L1 or their relative language proficiency on verbal fluency tasks) impact the segmentation process, and if the type of data examined (ERP vs. behavioral) can influence the conclusions one can draw regarding segmentation strategy adaptation. Interestingly, very different patterns of results were observed between ERPs and behavioral data. Specifically, individual differences in language experience (L1 or relative language dominance index) had a limited impact on the observed ERPs (of French trials only), but significantly affected the accuracy and reaction times associated with behavioral responses. In other words, participants showed signs of being capable of processing the heard speech in a native- or near-native like manner (ERP results), but nonetheless presented different final segmentation decisions (behavioral results).

Taken together, the results suggest that the ability to process the acoustic cues associated with L2 word segmentation in a native-like manner might not be sufficient to guarantee native-like final segmentation decisions. Thus, having the capacity to use an L2-specific segmentation strategy does not guarantee that listeners will use it systematically. For instance, a listener might be better at using the L2 strategy in some contexts than others (for example, when listening to high frequency vs low frequency words, or in a subset of grammatical constructions), which would lead to some trials being processed in a native-like manner (yielding correct response trials included in ERP analyses), and some trials being processed using an inappropriate segmentation strategy (yielding incorrect response trials not included in the ERP analyses but lowering behavioral accuracy scores). In this way, a listener could simultaneously show signs of being able to use the L2 appropriate speech segmentation strategy and yet present with lower accuracy scores in general. Possessing the ability to use an L2-specific segmentation strategy also does not guarantee that listeners will rely on it blindly to make their final segmentation decisions. For example, L2 listeners might systematically use the appropriate L2 segmentation strategies, but later revise their decision as if second-guessing their initial segmentation. Such a scenario would also lead to native-like ERPs to trials with correct responses regardless of lower accuracy scores overall. Of note, this scenario should also yield slower reaction times to correct responses and the elicitation of later ERP components associated with sentence reanalysis (e.g., P600, Friederici, 2002; Abada, 2010). Interestingly, the native English listeners of the present sample presented with native-like early ERPs (N1, N2, N4) in French trials, regardless of lower accuracy scores in general, and with slower reaction times to correct responses compared to native French listeners. Such a pattern of results might suggest that English listeners revised their initial segmentation of French sentences, (thus making their final segmentation decision non-native like) but could also simply be a sign of less efficient L2 processing. Unfortunately, the present design did not allow us to determine which of these interpretations better reflects the actual processes involved. Such investigation would require an analysis of the later ERPs associated with *incorrect* responses, which is not methodologically sound (one cannot presuppose the processes that lead to an incorrect response).

Aside from providing insights into L2 listeners' ability to adapt their segmentation strategies to the specifics of the L2, the present dataset provided interesting data about language processes in general, as summarized in Table 4. Below, we discuss the observed data patterns and their significance in greater detail.

### Interpretation of Event-Related Data Patterns

To our knowledge, no other experiment has investigated word segmentation using an audiovisual integration task. Thus, we based our ERP predictions on papers that only partly matched the present study. Here, two different sets of predictions were made. The first set of predictions was based on the speech segmentation literature (without visual stimuli) and focused on peaks within the N100 and late N200 time-windows. The second set of predictions was based on the audiovisual integration of single word stimuli (no word segmentation or bilinguals involved) and focused on peaks within the late N200 and N400 time-windows.

#### N1 Component

Based on the ERP literature on speech segmentation in English (Sanders and Neville, 2003a,b), we expected N1 amplitudes to vary as a function of sentence condition (syllable position within the word and stress) as well as participant L1. In the present dataset, the N1 varied as a function of sentence condition, but in a direction opposite to that observed by Sanders and Neville (2003a). Namely, trials from the one word condition (time-locked to a medial unstressed syllable) yielded more negative N1s than trials from the two word condition (time-locked to the onset of the second monosyllabic word). Of note, the amplitude of N1s during English trials was also characterized by an interaction between the sentence and picture conditions. This effect of picture condition on N1 could not be predicted based on the word segmentation literature, but mirrors other predicted effects (see discussion of the N2 below).

Interestingly, no effect of L1 or the relative language dominance index was observed on N1 amplitude in English trials, suggesting that the non-native listeners in the present sample processed English trials in a native- or near native-like manner, unlike the non-native listeners reported by Sanders and Neville (2003b). Of note, the L2 participants in this latter study differ from our participants on many levels, including their L1 (Japanese vs. French), their age of first exposure to English (average 12 vs. 6.61 years old), and the amount of time they were exposed to English spoken by native speakers. Further investigation is necessary to determine exactly what factors might explain the observed discrepancy.

Conversely, different patterns of N1 modulations were observed in French trials. For instance, unlike in English trials, the amplitude of N1 during French trials remained unaffected by sentence and picture conditions, like the N1s previously observed in the audiovisual integration literature (Yin et al., 2008; Hu et al., 2012). The absence of a sentence condition effect indicates that time-locking trials to word-initial or word-medial syllables had no significant impact on N1 amplitudes, which suggests that this N1 is likely related to audiovisual processing rather than speech segmentation *per se* (otherwise it should have been modulated at least by the sentence condition). Nonetheless, a significant effect of L1 was observed on mean N1 amplitudes where non-native listeners (English-L1 participants) presented with significantly lower N1 amplitudes than native listeners (French-L1 and simultaneous bilinguals). Interestingly, no significant effect of language dominance was observed, which suggests that the observed L1 effect is likely not a by-product of participants' lower French proficiency. Given that N1 has previously been associated with audiovisual integration and with lexical stress processing in English, such a result suggests that the early processes of English-L1 listeners might be affected by the absence of lexical stress in French trials (affecting their earliest stages of speech processing, leading to lower N1 amplitudes), but they nonetheless remain able to use French-appropriate segmentation cues as observed through the N2 and N4 components.

#### N2 Component

In terms of predictions for the late N200 time-windows, we expected modulations of N2 amplitude as a function of sentence and picture conditions. Similar to N1, trials from the one word condition (time-locked to a medial unstressed syllable) were expected to yield more negative N2s than trials from the two word condition (time-locked to the onset of monosyllabic words). Also, trials paired with a mismatching illustration were expected to yield more negative N2s than trials paired with a matching illustration. Interestingly, the observed N2 amplitudes were characterized by a significant interaction between conditions in both languages. Follow-up analyses demonstrated that the picture condition had the expected effect on N2 amplitude, at least to a certain degree, but that the sentence condition had the opposite effect, as observed for N1 amplitudes during English trials. Namely, the matching picture condition yielded lower amplitude N2s than the mismatching picture condition in the two word sentence condition, but not in the one word condition. In the one word condition, both picture conditions yielded N2s comparable to those elicited in the mismatching condition of the two word condition.

These results suggest that in correct response trials (trials included in the ERP averages), listeners had already determined that the first syllable of the two word condition (Eng. *key we* or Fr. *or loge*) consisted of a stand-alone word at the time of picture presentation (at the onset of *we* in English or the onset of *loge* in French). This allowed the listeners to detect the match between the auditory and visual stimuli (seeing a key while hearing *key we* or seeing gold bars while hearing *or loge*), leading to lower amplitude N2s. The lexical decision regarding the one bisyllabic word condition (Eng. *kiwi*, Fr. *horloge*) might not have been final at the time of picture presentation given that the second syllable had not yet been fully processed (Näätänen, 2001). In this context, all illustrations were interpreted as unrelated, regardless of the picture condition, which led to comparable N2 amplitudes across picture conditions. Furthermore, contrary to our predictions, no effect of L1 or relative language dominance was observed on N2 amplitude in either language. This suggests that the processes indexed by the N2 were used in a similar manner by both L1 and L2 listeners.

#### N4 Component

Finally, in terms of predictions for the N400 time-window, we expected modulations of the N4 as a function of the picture condition only, with mismatching pictures (i.e., matching the other sentence of the pair) yielding larger N4s than matching pictures. No specific predictions could be made regarding sentence conditions or bilingual listeners based on the extant literature. Nonetheless, a dampening or absence of the effect of picture condition among L2 listeners would be interpreted as a sign that they did not segment L2 speech in a native-like manner, leading to faulty segmentation, thereby hindering the observation of the link between the auditory stimulus and the visual display. As expected, we observed significant effects of picture condition in both languages, with mismatching pictures leading to larger amplitude N4s than matching pictures. This result suggests that participants had finalized their segmentation decision by that point and had accessed the lexical representation of the words in either condition (two monosyllabic words or one bisyllabic word condition), allowing for the detection of the match between the illustration and the heard stimuli (contrary to what was observed in earlier time-windows). Unexpectedly, a significant effect of sentence condition was also observed across language such that the one word condition consistently yielded larger amplitude N4s than the two word condition (no interaction between factors). This result suggests that the processing of the bisyllabic word of the one word sentence condition was more demanding in general than the processing of the monosyllabic words of the two word condition. Such an effect might reflect the fact that the bisyllabic words used in the present design are intrinsically harder to process than their monosyllabic equivalent, or could be a by-product of our using the same picture latency in both sentence conditions, which leads to the picture being presented *after* the first monosyllabic word of the two word condition, but *during* the bisyllabic word of the one word condition. Interestingly, participants' relative language dominance as indexed by English and French verbal fluency tasks was found to interact with the sentence condition in French trials only (not in English trials). Namely, higher relative language dominance indices (suggesting English dominance) were associated with similar N4 amplitudes across sentence conditions while lower relative language dominance indices (suggesting French dominance) were associated with lower N4 amplitudes for the two word condition compared to the one word condition. This pattern suggests that shorter French words are easier to process than longer French words for French dominant listeners, which is not the case for English dominant listeners. For them, the monosyllabic words of the two word condition are just as difficult to process as the bisyllabic words of the one word condition.

#### Summary of ERP Results

Taken together, these results showed that even if English and French have traditionally been associated with different segmentation strategies, they presented surprisingly similar ERP patterns in the present design. Moreover, the fact that individual differences in language experience (as indexed by participants' L1 or their relative language dominance index based on verbal fluency tasks) had no significant impact on the ERPs to correct English trials while having only a limited impact on the ERPs to correct French trials suggests that L2 proficiency might be an important factor in determining segmentation strategies. Looking at the proficiency measures presented in Table 1, one notices that the French-L1 participants of the present sample are highly proficient in English, some of them even performing much better on the English version of the verbal fluency task than on the French version (relative language dominance indices ranging from 0.56—suggesting French dominance, to 1.82—suggesting English dominance). Thus, the French-L1 participants of the present sample might have reached an L2 proficiency level allowing them to integrate an L2-specific segmentation strategy, which could explain why no effect of the relative language dominance index was observed on the ERPs for English trials.

On the other hand, all English-L1 participants performed better on the English version of the verbal fluency task than the French version (relative language dominance indices ranging from 1.08 to 2.47). Thus, the English-L1 participants of the present sample might not all have reached a high enough French-L2 proficiency level to allow them to learn to consistently adapt their segmentation strategy to that of French, which might explain why we did observe effects of relative language dominance index effects on the ERPs to French trials. Of note, given the L2 proficiency differences between the L1 groups in the current sample, one cannot completely rule out the possibility of directional language-specific effects, where it might be easier for French-L1 listeners to learn to segment speech in an English-like manner than the other way around, or that the language dominance index is simply not specific enough to capture variations in English proficiency. Nonetheless, these results show that the native French speakers of the present sample *were able to learn* to use L2-specific segmentation strategies, relying on lexical stress perception in a native-like (English) manner.

### Interpretation of Behavioral Results

Before reviewing the details of behavioral scores across languages, it is important to remember that none of the participant groups attained perfect accuracy. This might be partly due to stimuli characteristics, but it nonetheless reminds us that speech processing is not as automatic and stable as we like to think. We are examining segmentation strategies that might sometimes not work as expected, even for native listeners, or might need to be reviewed in light of communicative context.

During English trials, a significant interaction was observed between L1 and picture condition in response accuracy. Namely, native French speakers (French-L1 and simultaneous bilinguals) were as accurate as English-L1 listeners in recognizing the link between the heard sentence and the matching picture (saying that the picture of a key is related to the two word condition—*key we*, or saying that the picture of a kiwi is related to the one word condition—*kiwi*), but had a harder time determining that the picture corresponding to the other sentence of the pair *was not* related to the heard sentence (saying that the picture of a key *is not* related to the one word condition—*kiwi*, or saying that the picture of a kiwi *is not* related to the two word condition—*key we*). Thus, the segmentation of English trials by the native French listeners in the present sample seems not as consistent as the segmentation decisions made by English-L1 listeners. This pattern of results suggests that both interpretations (one word and two word) were still active at the time of picture presentation, indicating that the native French speakers in the present sample (French-L1 and simultaneous bilinguals) might not yet have reached a final segmentation decision.

Furthermore, an interaction between picture condition and L1 was also observed in the reaction times of simultaneous bilinguals during English trials. While the reaction times of every participant group were shorter for matching pictures than for pictures matching the other sentence of the pair, indicating that participants were more efficient at recognizing a matching condition than rejecting a mismatching condition (even English-L1 listeners), the difference between picture conditions was even greater for simultaneous bilinguals, suggesting that they might have to deal with more interference than participants who learned their L2 only later in life.

Interestingly, no effects of the relative language dominance index were observed on either response accuracy or reaction times for English trials, indicating that variations in English proficiency could not predict the observed pattern of behavioral results. Therefore, when considering only behavioral results, it seems that exposure to French from birth (as experienced by French-L1 participants and simultaneous bilinguals) had an impact on how efficiently listeners were able to use or rely on English's specific segmentation strategy regardless of their English proficiency.

Conversely, in French trials, both response accuracy and reaction times varied as a function of relative language proficiency as indexed by the relative language dominance index (regardless of participants' L1), with participants with higher index scores (at the English dominant end of the spectrum) producing slower and less accurate responses than participants with lower index scores (at the French dominant end of the spectrum). This finding suggests that even listeners who were first exposed to French only later in life show signs of being able to learn to segment these French utterances in a native-like manner as their French proficiency increases.

## Conclusion

Taken together, these ERP results showed that L2 participants are able to learn to use L2 segmentation strategies in a native-like manner, and behavioral findings (accuracy and reaction time) suggest that they might not do so as consistently as native listeners. Nevertheless, the present dataset allowed us to answer our research question by demonstrating that L2 participants did show signs of being able to adapt their segmentation strategies to the specifics of the L2 and that the quality of the adaptation varied as a function of listeners' language experience as indexed by their L1 and/or their relative language proficiency on verbal fluency tasks. Nonetheless, further studies will be required to investigate possible directional and/or language specific effects.

## Data Availability Statement

The datasets presented in this article are not readily available because data sharing was not part of the original ethics approval. Requests to access the datasets should be directed to annie.c.gilbert@mail.mcgill.ca.

## Ethics Statement

The studies involving human participants were reviewed and approved by Institutional Review Board of McGill University's Faculty of Medicine & Health Sciences. The patients/participants provided their written informed consent to participate in this study.

## Author Contributions

ACG and SRB designed the study in collaboration with SK, VLG, DK, DT, and NAP. ACG, KC, SK, MAW, and JGL carried out data collection. JGL preprocessed event-related data and ACG analyzed the data. All authors participated in data interpretation, critically reviewed the manuscript, and approved the submitted version.

## Funding

This work was supported by a team grant from the Fonds de Recherche du Québec—Société et Culture, and a generous donation from the Blema and Arnold Steinberg Family Foundation.

## Conflict of Interest

The authors declare that the research was conducted in the absence of any commercial or financial relationships that could be construed as a potential conflict of interest.

## Publisher's Note

All claims expressed in this article are solely those of the authors and do not necessarily represent those of their affiliated organizations, or those of the publisher, the editors and the reviewers. Any product that may be evaluated in this article, or claim that may be made by its manufacturer, is not guaranteed or endorsed by the publisher.
