# Supplementary material for: Spoken Word Segmentation in First and Second Language: When ERP and Behavioral Measures Diverge
Source: Front Psychol. 2021 Sep 17;12:705668. doi: 10.3389/fpsyg.2021.705668 (PMC8485064; doi:10.3389/fpsyg.2021.705668)
Supplement: Supplementary file 1 [file Data_Sheet_1.pdf]

Supplementary Material

Supplementary Table 1a: Detailed breakdown of included and rejected trials' accuracy rates as a function of sentence and picture conditions, French trials

	Included trials					Trials rejected based on reaction time					
	One word sentence condition (horloge)		Two word sentence condition (*or loge*)			One word sentence condition (*horloge*)		Two word sentence condition (*or loge*)			
	« Exact match » picture condition	« Match other » picture condition	« Exact match » picture condition	« Match other » picture condition	Total	« Exact match » picture condition	« Match other » picture condition	« Exact match » picture condition	« Match other » picture condition	Total	Grand Total
Number of trials	2394	2357	2336	2300	9387	145	182	204	241	772	10159
Average accuracy rate across participants (%)	82.77	90.50	62.02	80.02	78.87*	75.61	80.49	56.20	82.63	76.98*	
Standard deviation across participants	12.34	10.06	16.23	13.98	11.05*	24.15	26.17	32.14	22.93	20.54*	

Supplementary Table 1b: Detailed breakdown of included and rejected trials' accuracy rates as a function of sentence and picture conditions, English trials

	Included trials					Trials rejected based on reaction time					
	One word sentence condition (*kiwi*)		Two word sentence condition (*key we*)			One word sentence condition (*kiwi*)		Two word sentence condition (*key we*)			
	« Exact match » picture condition	« Match other » picture condition	« Exact match » picture condition	« Match other » picture condition	Total	« Exact match » picture condition	« Match other » picture condition	« Exact match » picture condition	« Match other » picture condition	Total	Grand total
Number of trials	2275	2205	2208	2187	8875	285	355	352	373	1365	10240
Average accuracy rate across participants (%)	90.44	79.67	75.38	66.07	78.05*	75.93	86.98	68.66	74.96	72.58*	
Standard deviation across participants	6.78	11.23	11.66	20.37	9.99*	30.75	20.07	26.90	22.21	25.56*	

* Computed across all "included" or "rejected" trials, without taking conditions into account. Given that some participants have a different number of trials in some conditions, these scores might differ from those that would be computed using mean accuracy rates per condition.

Supplementary Table 2: Effect of sentence condition (as a two-level deviation-coded categorical variable: one-word condition vs two-word condition) and picture condition (as a two-level categorical variable; "exact match" vs "match other") on mean ERP amplitude of N1, N2 and N4 in French trials.

	**ERP components**											
	**N1**				**N2**				**N4**			
**Fixed Effects**	*b*	*SE*	*t*	*p*	*b*	*SE*	*t*	*p*	*b*	*SE*	*t*	*p*
Intercept	-3.2401	0.3020	-10.728	< 0.0001	-7.9040	0.7785	-10.153	< 0.0001	-4.2096	0.7053	-5.969	< 0.0001
Sentence condition	0.3685	0.2706	1.362	0.1740	1.9095	0.4566	4.182	< 0.0001	1.7051	0.5806	2.937	0.00376
Picture♦ condition	-0.1752	0.1913	-0.916	0.3600	-1.0690	0.3228	-3.311	0.0011	-2.0944	0.4105	-5.102	< 0.0001
Sentence * Picture condition	-0.3196	0.3827	-0.835	0.4040	-1.5952	0.6457	-2.471	0.0144	-0.2687	0.8211	-0.327	0.74385
**Random Effects**	Variance				Variance				Variance			
	Intercept (*SD*)		Slope		Intercept (*SD*)		Slope		Intercept (*SD*)		Slope	
Participants	4.374 (2.091)		-		33.233 (5.765)		-		24.79 (4.979)		-	
Residual	4.394 (2.096)				6.253 (2.501)				10.11 (3.180)			
**Model fit**	Marginal		Conditional		Marginal		Conditional		Marginal		Conditional	
R^2	0.0028		0.5003		0.0188		0.8446		0.0471		0.7239	
AIC	2,215.942				1,313.792				1,384.587			
BIC	2,240.985				1,334.676				1,405.471			

♦ The "exact match" condition used as reference level, thus, the model tests the significance of the difference between "exact match" and "match other".

Supplementary Table 3: Effect of sentence condition (as a two-level deviation-coded categorical variable), picture condition (as a two-level categorical variable) and native language (as a three-level categorical variable) on mean ERP amplitude of N1, N2 and N4 in French trials.

	**ERP components**											
	**N1**				**N2**				**N4**			
**Fixed Effects**	*b*	*SE*	*t*	*p*	*b*	*SE*	*t*	*p*	*b*	*SE*	*t*	*p*
Intercept	-4.0311	0.4810	-8.381	< 0.0001	-8.0289	1.2709	-6.317	< 0.0001	-4.4353	1.1581	-3.830	0.0003
Sentence condition	0.0854	0.4379	0.195	0.8456	2.0092	0.7474	2.688	0.0079	1.6737	0.9504	1.761	0.0800
Picture condition♦	0.1925	0.3097	0.622	0.5345	-0.6979	0.5285	-1.320	0.1884	-1.9975	0.6720	-2.972	0.0034
Sentence * Picture conditions	-0.1358	0.6193	-0.219	0.8265	-1.7543	1.0570	-1.660	0.0988	-0.1770	1.3441	-0.132	0.8954
L1⋆												
Simultaneous bilinguals	0.8468	0.7509	1.128	0.2633	-0.6445	1.9842	-0.325	0.7464	0.0429	1.8081	0.024	0.9812
English-L1	1.6149	0.6962	2.320	0.0233	0.8479	1.8396	0.461	0.6465	0.6122	1.6764	0.365	0.7161
Sentence condition * L1												
Sentence condition * Simultaneous	0.1135	0.6837	0.166	0.8683	-0.1519	1.1669	-0.130	0.8966	0.7137	1.4838	0.481	0.6311
Sentence condition * English-L1	0.7226	0.6339	1.140	0.2550	-0.1690	1.0819	-0.156	0.8761	-0.4541	1.3757	-0.330	0.7417
Picture condition * L1												
Picture condition * Simultaneous	-0.5553	0.4835	-1.149	0.2514	-0.8597	0.8251	-1.042	0.2990	0.3384	1.0492	0.322	0.7475
Picture condition * English-L1	-0.6275	0.4482	-1.400	0.1623	-0.4054	0.7650	-0.530	0.5968	-0.5346	0.9728	-0.550	0.5834
Sentence condition * Picture condition * L1												
Sentence * Picture * Simultaneous	-0.2364	0.9669	-0.244	0.8070	-0.0164	1.6503	-0.010	0.9921	-0.7394	2.0985	-0.352	0.7250
Sentence * Picture * English-L1	-0.3450	0.8965	-0.385	0.7006	0.4669	1.5301	0.305	0.7606	0.3012	1.9456	0.155	0.8771

**Random Effects**	Variance		Variance		Variance	
	Intercept (*SD*)	Slope	Intercept (*SD*)	Slope	Intercept (*SD*)	Slope
Participants	4.218 (2.054)	-	33.938 (5.826)	-	25.65 (5.065)	-
Residual	4.411 (2.100)		6.425 (2.535)		10.39 (3.223)	
**Model fit**	Marginal	Conditional	Marginal	Conditional	Marginal	Conditional
R^2	0.0418	0.5102	0.0299	0.8456	0.0477	0.7255
AIC	2,217.810		1,311.481		1,380.556	
BIC	2,276.243		1,360.209		1,429.285	

♦ The "exact match" condition used as reference level, thus, the model tests the significance of the difference between "exact match" and "match other".

⋆ French-L1 used as reference level, thus, the model tests the significance of the difference between French-L1 and other L1 categories (i.e., difference between French-L1 and English-L1, difference between French-L1 and Simultaneous bilinguals).

Supplementary Table 4: Effect of sentence condition (as a two-level deviation-coded categorical variable: one-word condition vs two-word condition), picture condition (as a two-level categorical variable; "exact match" vs "match other") and relative language dominance (as a scaled continuous variable) on mean ERP amplitude of N1, N2 and N4 in French trials.

	ERP components											
	N1				N2				N4			
**Fixed Effects**	*b*	*SE*	*t*	*p*	*b*	*SE*	*t*	*p*	*b*	*SE*	*t*	*p*
Intercept	-3.2401	0.3007	-10.774	< 0.0001	-7.9040	0.7842	-10.080	< 0.0001	-4.2096	0.7020	-5.997	< 0.0001
Sentence condition	0.3685	0.2710	1.360	0.1750	1.9095	0.4578	4.171	< 0.0001	1.7051	0.5767	2.957	0.00354
Picture condition♦	-0.1752	0.1916	-0.914	0.3610	-1.0690	0.3237	-3.302	0.0012	-2.0944	0.4078	-5.136	< 0.0001
Sentence * Picture conditions	-0.3196	0.3832	-0.834	0.4050	-1.5952	0.6475	-2.464	0.0147	-0.2687	0.8156	-0.329	0.74218
Relative language dominance★	0.4737	0.3010	1.574	0.1200	-0.2371	0.7858	-0.302	0.7638	-0.8871	0.7035	-1.261	0.21156
Sentence condition * Rel. lang. dominance	0.1496	0.2713	0.552	0.5820	-0.2478	0.4588	-0.540	0.5898	-1.2989	0.5779	-2.248	0.02586
Picture condition * Rel. lang. dominance	-0.2307	0.1918	-1.203	0.2300	-0.0606	0.3244	-0.187	0.8521	0.1092	0.4086	0.267	0.78952
Sentence * Picture * Rel. lang. dominance	-0.2468	0.3836	-0.643	0.5200	-0.3509	0.6488	-0.541	0.5893	0.9921	0.8173	1.214	0.22643
**Random Effects**	Variance				Variance				Variance			
	Intercept (*SD*)		Slope		Intercept (*SD*)		Slope		Intercept (*SD*)		Slope	
Participants	4.325 (2.080)		-		33.751 (5.810)		-		24.579 (4.958)		-	
Residual	4.406 (2.099)				6.288 (2.508)				9.977 (3.159)			
**Model fit**	Marginal		Conditional		Marginal		Conditional		Marginal		Conditional	
R^2	0.0192		0.5051		0.0216		0.8463		0.0711		0.7318	
AIC	2,224.168				1,318.193				1,383.095			
BIC	2,265.906				1,352.999				1,417.901			

♦ The "exact match" condition used as reference level, thus, the model tests the significance of the difference between "exact match" and "match other".

★ Relative language dominance index > 1 = more proficient in English than in French, relative language dominance index < 1 = more proficient in French than English.

Supplementary Table 5: Effect of sentence condition (as a two-level deviation-coded categorical variable: one-word condition vs two-word condition) and picture condition (as a two-level categorical variable; "exact match" vs "match other") on behavioural results to French trials. Models also include (log) word frequency to control for its possible effect on participant responses.

	Accuracy				Response times to correct trials			
**Fixed Effects**	*b*	*SE*	*z*	*p*	*b*	*SE*	*t*	*p*
Intercept	-0.2729	0.2204	-1.239	0.2155	1073.16	42.218	25.420	< 0.0001
Word frequency (log)	0.5538	0.0361	15.359	< 0.0001	-20.538	3.204	-6.411	< 0.0001
Sentence condition	-1.8479	0.0908	-20.354	< 0.0001	59.220	9.993	5.926	< 0.0001
Picture condition♦	0.9087	0.1914	4.747	< 0.0001	38.380	14.076	2.727	0.0098
Sentence * Picture conditions	0.3260	0.1284	2.538	0.0112	-7.932	12.857	-0.617	0.5373

**Random Effects**	Variance		Variance	
	Intercept (*SD*)	Slope†	Intercept (*SD*)	Slope†
Participants	0.7188 (0.8478)	-	86,128 (293.48)	-
Items (sentence pairs)	1.0163 (1.0081)	1.2830 (1.1327)	9,941 (99.71)	6,259 (79.11)
Residual	-		67,995 (260.76)	
**Model fit**	Marginal	Conditional	Marginal	Conditional
R^2	0.1320	0.3212	0.0100	0.5857
AIC	7,395.153		98,454.11	
BIC	7,459.048		98,522.67	

♦ The "exact match" condition was used as reference level, thus, the model tests the significance of the difference between "exact match" and "match other".

† Random Slope adjustments were done on Picture condition across items (sentence pairs).

Supplementary Table 6: Effect of sentence condition (as a two-level deviation-coded categorical variable: one-word condition vs two-word condition), picture condition (as a two-level categorical variable; "exact match" vs "match other") and native language (as a three-level categorical variable; English-L1, simultaneous bilingual, and French-L1) on behavioural results to French trials. Models also include (log) word frequency to control for its possible effect on participant responses.

	**Accuracy**				**Response times to correct trials**			
**Fixed Effects**	*b*	*SE*	*z*	*p*	*b*	*SE*	*t*	*p*
Intercept	0.0799	0.2331	0.342	0.7320	971.186	62.394	15.565	< 0.0001
Word frequency (log)	0.5314	0.0345	15.391	< 0.0001	-20.474	3.203	-6.393	< 0.0001
Sentence condition	-1.8609	0.1390	-13.383	< 0.0001	56.847	14.743	3.856	0.0001
Picture♦ condition	0.8668	0.1053	8.231	< 0.0001	28.257	15.948	1.772	0.0816
Sentence * Picture♦ conditions	0.3626	0.2099	1.728	0.0841	-34.185	19.671	-1.738	0.0823
L1★								
Simultaneous bilinguals	-0.2593	0.2776	-0.934	0.3503	99.906	96.863	1.031	0.3066
English-L1	-0.7620	0.2384	-3.196	0.0014	199.909	83.453	2.395	0.0198
Sentence condition * L1★								
Sentence condition * Simultaneous	-0.0770	0.2122	-0.363	0.7168	12.089	23.860	0.507	0.6124
Sentence condition * English-L1	0.2664	0.1780	1.497	0.1344	-2.044	21.433	-0.095	0.9240
Picture♦ condition * L1★								
Picture condition * Simultaneous	-0.1678	0.1654	-1.014	0.3105	21.559	16.384	1.316	0.1883
Picture condition * English-L1	0.2197	0.1409	1.560	0.1189	15.009	14.509	1.034	0.3010
Sentence condition * Picture♦ condition * L1★								
Sentence * Picture * Simultaneous	0.0297	0.3301	0.090	0.9284	61.640	32.713	1.884	0.0596
Sentence * Picture * English-L1	-0.1813	0.2808	-0.645	0.5186	35.426	28.924	1.225	0.2207
**Random Effects**	Variance				Variance			
	Intercept (*SD*)		Slope		Intercept (*SD*)		Slope†	
Participants	0.5668 (0.7528)		-		80,404 (283.56)		-	
Items (sentence pairs)	0.6529 (0.8080)				9,951 (99.76)		6,276 (79.22)	
Residual	-				67,924 (260.62)			
**Model fit**	Marginal		Conditional		Marginal		Conditional	
R^2	0.1459		0.2927		0.0591		0.5922	
AIC	7,638.513				98,386.48			
BIC	7,745.004				98,506.89			

♦ The "exact match" condition used as reference level.

★ French-L1 used as reference level, thus, the model tests the significance of the difference between French-L1 and other L1 categories (i.e., difference between French-L1 and English-L1, difference between French-L1 and Simultaneous bilinguals).

† Random Slope adjustments were done on Picture condition across items (sentence pairs).

Supplementary Table 7: Effect of sentence condition (as a two-level deviation-coded categorical variable: one-word condition vs two-word condition), picture condition (as a two-level categorical variable; "exact match" vs "match other") and relative language dominance (as a scaled continuous variable) on behavioural results to French trials. Models also include (log) word frequency to control for its possible effect on participant responses.

	Accuracy				Response times to correct trials			
**Fixed Effects**	*b*	*SE*	*z*	*p*	*b*	*SE*	*t*	*p*
Intercept	-0.2696	0.1836	-1.469	0.142	1065.7418	41.6877	25.565	< 0.0001
Word frequency (log)	0.5311	0.0345	15.394	< 0.0001	-20.7114	3.2052	-6.462	< 0.0001
Sentence condition	-1.7698	0.0878	-20.147	< 0.0001	59.4931	10.0031	5.947	< 0.0001
Picture condition♦	0.9373	0.0634	14.775	< 0.0001	38.4823	14.0981	2.730	0.0098
Sentence * Picture conditions	0.2564	0.1261	2.034	0.042	-8.0876	12.8639	-0.629	0.5296
Relative language dominance★	-0.4385	0.0921	-4.759	< 0.0001	69.5671	35.0867	1.983	0.0520
Sentence condition * Relative language dominance	0.0903	0.0739	1.222	0.222	-0.0908	9.4170	-0.010	0.9923
Picture condition * Relative language dominance	-0.0523	0.0569	-0.920	0.358	-2.6428	6.3954	-0.413	0.6795
Sentence * Picture condition * Rel. lang. dominance	0.0226	0.1133	0.199	0.842	17.3636	12.7199	1.365	0.1723

**Random Effects**	Variance		Variance	
	Intercept (*SD*)	Slope	Intercept (*SD*)	Slope†
Participants	0.4355 (0.6599)	-	82,365 (286.99)	-
Items (sentence pairs)	0.6504 (0.8064)	-	9,954 (99.77)	6,283 (79.26)
Residual	-		67,978 (260.73)	
**Model fit**	Marginal	Conditional	Marginal	Conditional
R^2	0.1632	0.2937	0.0367	0.5875
AIC	7,622.824		98,427.12	
BIC	7,700.917		98,523.11	

♦ The "exact match" condition used as reference level.

★ Relative language dominance index > 1 = more proficient in English than in French, relative language dominance index < 1 = more proficient in French than English.

† Random Slope adjustments were done on Picture condition across items (sentence pairs).

Supplementary Table 8: Effect of sentence condition (as a two-level deviation-coded categorical variable: one-word condition vs two-word condition) and picture condition (as a two-level categorical variable; “exact match” vs “match other”) on mean ERP amplitude of N1, N2 and N4 in English trials.

	**ERP components**											
	**N1**				**N2**				**N4**			
**Fixed Effects**	*b*	*SE*	*t*	*p*	*b*	*SE*	*t*	*p*	*b*	*SE*	*t*	*p*
Intercept	-3.5746	0.2947	-12.130	< 0.0001	-5.8566	0.5920	-9.893	< 0.0001	-3.7386	0.5292	-7.065	< 0.0001
Sentence condition	1.0452	0.2816	3.711	0.0002	2.0368	0.5688	3.581	0.0004	1.4036	0.5974	2.350	0.0193
Picture♦ condition	-0.2022	0.1997	-1.012	0.3119	-0.9273	0.4033	-2.299	0.0220	-1.8371	0.4236	-4.337	< 0.0001
Sentence * Picture condition	-1.1999	0.3994	-3.004	0.0028	-2.5665	0.8066	-3.182	0.0016	-0.9386	0.8472	-1.108	0.2686
**Random Effects**	Variance				Variance				Variance			
	Intercept (*SD*)		Slope		Intercept (*SD*)		Slope		Intercept (*SD*)		Slope	
Participants	3.886 (1.971)		-		15.63 (3.954)		-		11.07 (3.326)		-	
Residual	4.601 (2.145)				18.76 (4.332)				20.70 (4.550)			
**Model fit**	Marginal		Conditional		Marginal		Conditional		Marginal		Conditional	
R^2	0.0174		0.4674		0.0219		0.4665		0.0341		0.3706	
AIC	2,146.264				2,789.416				2,813.118			
BIC	2,171.077				2,814.230				2,837.932			

♦ The “exact match” condition used as reference level, thus, the model tests the significance of the difference between “exact match” and “match other”.

Supplementary Table 9: Effect of sentence condition (as a two-level deviation-coded categorical variable), picture condition (as a two-level categorical variable) and native language (as a three-level categorical variable) on mean ERP amplitude of N1, N2 and N4 in English trials.

	**ERP components**											
	**N1**				**N2**				**N4**			
**Fixed Effects**	*b*	*SE*	*t*	*p*	*b*	*SE*	*t*	*p*	*b*	*SE*	*t*	*p*
Intercept	-2.9749	0.4857	-6.125	< 0.0001	-4.9722	0.9790	-5.079	< 0.0001	-3.4980	0.8800	-3.975	0.0002
Sentence condition	1.4370	0.4701	3.057	0.0024	2.2086	0.9509	2.323	0.0207	1.3179	0.9964	1.323	0.1867
Picture condition♦	-0.1559	0.3324	-0.469	0.6394	-0.6493	0.6724	-0.966	0.3348	-1.4593	0.7046	-2.071	0.0390
Sentence * Picture conditions	-1.5730	0.6648	-2.366	0.0185	-2.8871	1.3448	-2.147	0.0324	-1.0402	1.4091	-0.738	0.4608
L1★												
Simultaneous bilinguals	-0.8168	0.7386	-1.106	0.2726	-0.8892	1.4887	-0.597	0.5522	0.2205	1.3382	0.165	0.8695
French-L1	-1.0339	0.6869	-1.505	0.1368	-1.7652	1.3845	-1.275	0.2065	-0.8326	1.2445	-0.669	0.5055
Sentence condition * L1												
Sentence condition * Simultaneous	-0.2566	0.7149	-0.359	0.7198	0.2461	1.4460	0.170	0.8650	1.1845	1.5152	0.782	0.4348
Sentence condition * French-L1	-0.8865	0.6648	-1.333	0.1832	-0.6617	1.3448	-0.492	0.6230	-0.6657	1.4091	-0.472	0.6369
Picture condition * L1												
Picture condition * Simultaneous	0.0464	0.5055	0.092	0.9269	-0.0564	1.0225	-0.055	0.9560	-0.0609	1.0714	-0.057	0.9547
Picture condition * French-L1	-0.1641	0.4720	-0.348	0.7283	-0.7386	0.9547	-0.774	0.4396	-1.0042	1.0003	-1.004	0.3160
Sentence condition * Picture condition * L1												
Sentence * Picture * Simultaneous	-0.0508	1.0110	-0.050	0.9600	0.2456	2.0450	0.120	0.9045	-1.4094	2.1428	-0.658	0.5111
Sentence * Picture * French-L1	1.0679	0.9439	1.131	0.2586	0.6708	1.9093	0.351	0.7255	1.3400	2.0006	0.670	0.5034
**Random Effects**	Variance				Variance				Variance			
	Intercept (*SD*)		Slope		Intercept (*SD*)		Slope		Intercept (*SD*)		Slope	
Participants	3.794 (1.948)		-		15.38 (3.922)		-		11.05 (3.324)		-	
Residual	4.641 (2.154)				18.99 (4.357)				20.85 (4.566)			
**Model fit**	Marginal		Conditional		Marginal		Conditional		Marginal		Conditional	
R^2	0.0466		0.4755		0.0457		0.4728		0.0514		0.3800	
AIC	2,150.311				2,783.894				2,806.407			
BIC	2,208.208				2,841.792				2,864.305			

♦ The "exact match" condition used as reference level, thus, the model tests the significance of the difference between "exact match" and "match other".

★ English-L1 used as reference level, thus, the model tests the significance of the difference between English-L1 and other L1 categories (i.e., difference between English-L1 and French-L1, difference between English-L1 and Simultaneous bilinguals).

Supplementary Table 10: Effect of sentence condition (as a two-level deviation-coded categorical variable: one-word condition vs two-word condition), picture condition (as a two-level categorical variable; "exact match" vs "match other") and relative language dominance (as a scaled continuous variable) on mean ERP amplitude of N1, N2 and N4 in English trials.

	ERP components											
	**N1**				**N2**				**N4**			
**Fixed Effects**	*b*	*SE*	*t*	*p*	*b*	*SE*	*t*	*p*	*b*	*SE*	*t*	*p*
Intercept	-3.5746	0.2967	-12.048	< 0.0001	-5.8566	0.5902	-9.924	< 0.0001	-3.7386	0.5331	-7.013	< 0.0001
Sentence condition	1.0452	0.2816	3.712	0.0002	2.0368	0.5694	3.577	0.0004	1.4036	0.5970	2.351	0.0192
Picture condition♦	-0.2039	0.1997	-1.021	0.3078	-0.9325	0.4038	-2.309	0.0214	-1.8408	0.4234	-4.348	< 0.0001
Sentence * Picture conditions	-1.2033	0.3994	-3.013	0.0028	-2.5770	0.8076	-3.191	0.0015	-0.9460	0.8467	-1.117	0.2646
Relative language dominance⋆	-0.0369	0.2970	-0.124	0.9016	0.3823	0.5908	0.647	0.5196	-0.3164	0.5336	-0.593	0.5550
Sentence condition * Rel. lang. dominance	0.3211	0.2819	1.139	0.2554	0.3042	0.5700	0.534	0.5939	0.4373	0.5977	0.732	0.4648
Picture condition * Rel. lang. dominance	0.2731	0.2000	1.365	0.1729	0.5471	0.4045	1.353	0.1770	0.7194	0.4241	1.696	0.0906
Sentence * Picture * Rel. lang. dominance	-0.3500	0.4001	-0.875	0.3822	-0.1933	0.8090	-0.239	0.8113	-0.5811	0.8483	-0.685	0.4937
**Random Effects**	Variance				Variance				Variance			
	Intercept (*SD*)		Slope		Intercept (*SD*)		Slope		Intercept (*SD*)		Slope	
Participants	3.956 (1.989)		-		15.50 (3.937)		-		11.31 (3.363)		-	
Residual	4.599 (2.144)				18.81 (4.336)				20.67 (4.547)			
**Model fit**	Marginal		Conditional		Marginal		Conditional		Marginal		Conditional	
R^2	0.0220		0.4742		0.0361		0.4716		0.0385		0.3785	
AIC	2,154.435				2,791.757				2,815.476			
BIC	2,195.791				2,833.112				2,856.832			

♦ The "exact match" condition used as reference level, thus, the model tests the significance of the difference between "exact match" and "match other".

⋆ Relative language dominance index > 1 = more proficient in English than in French, relative language dominance index < 1 = more proficient in French than English.

Supplementary Table 11: Effect of sentence condition (as a two-level deviation-coded categorical variable: one-word condition vs two-word condition) and picture condition (as a two-level categorical variable; “exact match” vs “match other”) on behavioural results to English trials. Models also include (log) word frequency to control for its possible effect on participant responses.

	Accuracy				Response times to correct trials			
**Fixed Effects**	*b*	*SE*	*z*	*p*	*b*	*SE*	*t*	*p*
Intercept	1.8056	0.2074	8.707	< 0.0001	1050.9456	42.0732	24.979	< 0.0001
Word frequency (log)	0.1788	0.0209	8.561	< 0.0001	-0.3555	2.2677	-0.157	0.8754
Sentence condition	-1.9674	0.1207	-16.295	< 0.0001	37.9353	11.2352	3.376	0.0007
Picture condition♦	-0.8593	0.2017	-4.259	< 0.0001	104.3690	11.5215	9.059	< 0.0001
Sentence * Picture conditions	0.4587	0.1331	3.446	0.0006	-20.0453	14.3386	-1.398	0.1622
**Random Effects**	Variance				Variance			
	Intercept (*SD*)		Slope†		Intercept (*SD*)		Slope†	
Participants	0.5411 (0.7356)		-		86,889 (294.77)		-	
Items (sentence pairs)	1.1722 (1.0827)		1.4212 (1.1922)		11,896 (109.07)		3,163 (56.24)	
Residual	-				76,145 (275.94)			
**Model fit**	Marginal		Conditional		Marginal		Conditional	
R^2	0.0926		0.3023		0.0169		0.5674	
	7,050.594				89,777.25			
	7,113.736				89,844.81			

♦ The “exact match” condition was used as reference level, thus, the model tests the significance of the difference between “exact match” and “match other”.

† Random Slope adjustments were done on Picture condition across items (sentence pairs).

Supplementary Table 12: Effect of sentence condition (as a two-level deviation-coded categorical variable: one-word condition vs two-word condition), picture condition (as a two-level categorical variable; “exact match” vs “match other”) and native language (as a three-level categorical variable; English-L1, simultaneous bilingual, and French-L1) on behavioural results to English trials. Models also include (log) word frequency to control for its possible effect on participant responses.

	**Accuracy**				**Response times to correct trials**			
**Fixed Effects**	*b*	*SE*	*z*	*p*	*b*	*SE*	*t*	*p*
Intercept	1.8005	0.2152	8.368	< 0.0001	1121.6965	63.3956	17.694	< 0.0001
Word frequency (log)	0.1834	0.0203	9.020	< 0.0001	-0.3583	2.2673	-0.158	0.8745
Sentence condition	-1.9417	0.1766	-10.997	< 0.0001	39.1899	16.3927	2.391	0.0169
Picture♦ condition	-0.5448	0.1066	-5.112	< 0.0001	85.8184	14.4055	5.957	< 0.0001
Sentence * Picture♦ conditions	0.4673	0.2130	2.194	0.0282	-9.6560	22.3784	-0.431	0.6661
L1★								
Simultaneous bilinguals	-0.1341	0.2547	-0.527	0.5984	-109.4528	100.1330	-1.093	0.2788
French-L1	-0.1605	0.2217	-0.724	0.4691	-120.5438	86.9087	-1.387	0.1707
Sentence condition * L1★								
Sentence condition * Simultaneous	-0.0190	0.2567	-0.074	0.9411	1.8877	24.6889	0.076	0.9391
Sentence condition * French-L1	-0.0004	0.2238	-0.002	0.9984	-4.8605	21.7128	-0.224	0.8229
Picture♦ condition * L1★								
Picture condition * Simultaneous	-0.3775	0.1655	-2.280	0.0226	48.9032	18.3226	2.669	0.0076
Picture condition * French-L1	-0.4846	0.1454	-3.333	0.0009	19.4725	16.0902	1.210	0.2263
Sentence condition * Picture♦ condition * L1★								
Sentence * Picture * Simultaneous	0.1211	0.3308	0.366	0.7143	-11.2797	36.4963	-0.309	0.7573
Sentence * Picture * French-L1	0.0082	0.2902	0.028	0.9774	-20.4658	32.0435	-0.639	0.5231
**Random Effects**	Variance				Variance			
	Intercept (*SD*)		Slope†		Intercept (*SD*)		Slope†	
Participants	0.4183 (0.6468)		-		87,285 (295.44)		-	
Items (sentence pairs)	0.8126 (0.9014)		-		11,904 (109.10)		3,206 (56.62)	
Residual	-				76,111 (275.88)			
**Model fit**	Marginal		Conditional		Marginal		Conditional	
R^2	0.0998		0.2587		0.0322		0.5752	
AIC	7,374.772				89,714.03			
BIC	7,480.009				89,835.64			

♦ The “exact match” condition used as reference level.

★ English-L1 used as reference level, thus, the model tests the significance of the difference between English-L1 and other L1 categories (i.e., difference between English-L1 and French-L1, difference between English-L1 and Simultaneous bilinguals).

† Random Slope adjustments were done on Picture condition across items (sentence pairs).

Supplementary Table 13: Effect of sentence condition (as a two-level deviation-coded categorical variable: one-word condition vs two-word condition), picture condition (as a two-level categorical variable; “exact match” vs “match other”) and relative language dominance (as a scaled continuous variable) on behavioural results to English trials. Models also include (log) word frequency to control for its possible effect on participant responses.

	**Accuracy**				**Response times to correct trials**			
**Fixed Effects**	*b*	*SE*	*z*	*p*	*b*	*SE*	*t*	*p*
Intercept	1.7186	0.1779	9.661	< 0.0001	1051.0509	42.1137	24.957	< 0.0001
Word frequency (log)	0.1828	0.0203	9.007	< 0.0001	-0.3498	2.2683	-0.154	0.8774
Sentence condition	-1.9472	0.1189	-16.376	< 0.0001	37.9031	11.2374	3.373	0.0007
Picture condition♦	-0.8350	0.0632	-13.220	< 0.0001	104.3427	11.5109	9.065	< 0.0001
Sentence * Picture conditions	0.5086	0.1257	4.047	< 0.0001	-20.0960	14.3414	-1.401	0.1612
Relative language dominance★	0.0330	0.0988	0.334	0.7390	35.3024	37.6740	0.937	0.3525
Sentence condition * Relative language dominance	0.0244	0.0931	0.262	0.7940	-1.8539	9.4797	-0.196	0.8450
Picture condition * Relative language dominance	0.0632	0.0606	1.041	0.2980	-0.9800	7.1081	-0.138	0.8903
Sentence * Picture * Rel. lang. dominance	-0.0484	0.1211	-0.400	0.6890	10.7177	14.0968	0.760	0.4471
**Random Effects**	Variance				Variance			
	Intercept (*SD*)		Slope†		Intercept (*SD*)		Slope†	
Participants	0.4498 (0.6707)		-		87,100 (295.13)		-	
Items (sentence pairs)	0.8091 (0.8995)		-		11,892 (109.05)		3,152 (56.15)	
Residual	-				76,172 (275.99)			
**Model fit**	Marginal		Conditional		Marginal		Conditional	
R^2	0.0951		0.2578		0.0238		0.5708	
AIC	7,383.027				89,755.88			
BIC	7,460.200				89,850.46			

♦ The “exact match” condition used as reference level.

★ Relative language dominance index > 1 = more proficient in English than in French, relative language dominance index < 1 = more proficient in French than English.

† Random Slope adjustments were done on Picture condition across items (sentence pairs).
